# Natural Antimicrobial Peptides and Their Synthetic Analogues for Effective Oral Microflora Control and Oral Infection Treatment—The Role of Ceragenins in the Development of New Therapeutic Methods

**DOI:** 10.3390/ph17121725

**Published:** 2024-12-20

**Authors:** Michał Czarnowski, Urszula Wnorowska, Milena Łuckiewicz, Ewelina Dargiewicz, Jakub Spałek, Sławomir Okła, Beata Sawczuk, Paul B. Savage, Robert Bucki, Ewelina Piktel

**Affiliations:** 1 Department of Medical Microbiology and Nanobiomedical Engineering, Medical University of Bialystok, 15-222 Bialystok, Poland; flaps.czarnowski@gmail.com (M.C.); urszula.wnorowska@umb.edu.pl (U.W.); 2 Independent Laboratory of Nanomedicine, Medical University of Bialystok, 15-222 Bialystok, Poland; milenaluckiewicz@gmail.com; 3 Department of Orthodontics, Medical University of Bialystok, 15-269 Bialystok, Poland; ewelina.dargiewicz@umb.edu.pl; 4 Institute of Medical Sciences, Collegium Medicum, Jan Kochanowski University of Kielce, 25-369 Kielce, Poland; jakub.spalek@ujk.edu.pl (J.S.); slawomir.okla@ujk.edu.pl (S.O.); 5 Department of Prosthodontics, Medical University of Bialystok, 15-276 Bialystok, Poland; beata.sawczuk@wp.pl; 6 Department of Chemistry & Biochemistry, Brigham Young University, Provo, UT 84602, USA; pbsavage@chem.byu.edu

**Keywords:** antimicrobial peptides, oral cavity, ceragenins

## Abstract

Oral diseases, both acute and chronic, of infectious or non-infectious etiology, represent some of the most serious medical problems in dentistry. Data from the literature increasingly indicate that changes in the oral microbiome, and therefore, the overgrowing of pathological microflora, lead to a variety of oral-localized medical conditions such as caries, gingivitis, and periodontitis. In recent years, compelling research has been devoted to the use of natural antimicrobial peptides as therapeutic agents in the possible treatment of oral diseases. This review focuses on the potential of ceragenins (CSAs), which are lipid analogs of natural antimicrobial peptides, as molecules for the development of new methods for the prevention and treatment of oral diseases. Studies to date indicate that ceragenins, with their spectrum of multidirectional biological activities, including antimicrobial, tissue regeneration-stimulating, anti-inflammatory, and immunomodulatory properties, are strong candidates for further development of oral formulations. However, many of the beneficial properties of ceragenins require confirmation in experimental conditions reproducing the oral environment to fully determine their application potential. Their transition to practical use also requires more advanced testing of these molecules in clinical trials, which have only been conducted in limited numbers to date.

## 1. Introduction

The primary function of the oral microbiota, which is a diverse and complex community of microorganisms that reside in the oral cavity, is to act as a barrier that prevents colonization by potentially harmful pathogens and, thus, plays an important role in the maintenance of the health of the oral cavity environment [1]. The intricate complexity of the oral microbiota becomes evident when considering its extensive diversity. It is widely acknowledged that the oral cavity serves as a reservoir for a wide range of microorganisms, with an estimated number of ~1000 species, which makes it the second most complex microbiota in the human body, surpassed only by the gut microbiota in terms of its diversity and composition [1,2,3]. The oral microbiome consists of a diverse range of microorganisms, including bacteria, archaea, fungi, viruses, and protozoa, inhabiting different areas and surfaces in the oral cavity, such as the teeth and soft tissues of the oral mucosa. Furthermore, it is crucial to acknowledge that the oral microbiome can exist in many forms, either as dispersed organisms in saliva or as a biofilm, including plaque, that adheres to oral surfaces. The phylogenetic makeup of microbial communities also undergoes temporal evolution and modifications due to natural and pathological processes [4]. However, it has been proposed that an imbalance in the composition of the oral microbiota may contribute to the occurrence of local and systemic diseases, including squamous cell carcinoma of the head and neck [4], rheumatoid arthritis [5], and hypertension [6]. These observations highlight the importance of understanding and effectively managing oral microbiota, as they reflect the status of the host immunological system and metabolism to a certain degree [7] since oral homeostasis disturbances in bacterial infections depend on several components, which not only include the individual pathogen’s characteristics, but also diet and host factors [8]. The subtle balance between commensal and pathogenic oral microorganisms is assured primarily by a spectrum of immune molecules, namely antimicrobial peptides (AMPs)—short (generally <100 amino acids) cationic molecules, which control the oral microbiota’s composition quantitatively and qualitatively [9]. AMPs widely exist in nature and have been identified in large numbers in vertebrates, invertebrates, plants, and bacteria [10]. They are an essential component of all living organisms’ innate immunity, augmenting the host’s physical and chemical barriers, including skin and mucous membranes. Although they are placed into different classes due to amino acid composition, size, and conformational differences (including anionic peptides, linear cationic α-helical peptides, and cationic peptides enriched for specific amino acids) [11], they display a variety of distinctive and overlapping biological functions, allowing them to modulate the antimicrobial capability of the host via antibacterial, antiparasitic, antifungal, and antiviral activities. In this aspect, cathelicidins, including human cathelicidin LL-37, and defensins are recognized as the most vital contributors to oral hemostasis maintenance [11].

This review focuses on the significance of AMPs for oral health and disease prevention and describes how AMPs contribute to maintaining oral homeostasis by providing an immediate and effective defense against microbial pathogens, modulating immune responses, and preserving the balance of the oral microbiome. The clinical utility of synthetic analogs of AMPs, particularly lipid-based analogs (so-called ceragenins, CSAs, i.e., cationic steroid antibiotics), and their potential as therapeutic agents for the prevention and treatment of oral infections and diseases, described in already established and most up-to-date reports, will be given special consideration herein. The increased stability of ceragenins, specificity of their activity, decreased likelihood of resistance development, antibiofilm characteristics, and immunomodulatory qualities render them advantageous alternatives to current therapeutic approaches.

## 2. Oral Microbiota’s Composition and Diversity—The Alterations in Oral Microbiota That Occur with Oral Illnesses

An alteration in the symbiotic connection between the oral microbial community and the host may be the result of a disturbance in the composition and function of the indigenous oral microbiome. This disturbance may have repercussions for the oral and general health of the individual. By disrupting the delicate equilibrium that exists among the microorganisms within the host—a phenomenon known as dysbiosis—pathogenic bacteria can exert their capacity to cause illness and establish pathological conditions [12]. While it is generally difficult to ascertain which oral bacteria are important for maintaining health and which are associated with oral diseases, there are indications that healthy individuals’ oral microflora consists primarily of aerobes and obligate anaerobes belonging to the following genera: *Streptococcus, Veillionella*, *Actinomyces*, *Neisseria*, *Candida* spp., and *Rothia mucilaginosa* [13,14]. At the same time, a disrupted abundance of *Fusobacterium*, *Neisseria*, *Streptococcus mutans*, *Porphyromonas gingivalis*, and *Lactobacillus* spp. is associated with dental diseases [15,16]; therefore, daily and therapeutic maintenance of oral microbiome hemostasis is critical for proper oral hygiene.

Chronic bacterial infections may cause dental caries, widely acknowledged as the most prevalent oral disease. Changes in the populations of oral bacteria in individuals with and without caries have been correlated to adverse outcomes, even for very young children [17], including children as young as three years and before clinical caries detection [18]. In one report, Zheng et al. showed that there was a greater presence of *Streptococcus mutans* in children with a high number of cavities compared to those who were cavity-free. At the same time, there was no significant difference between the two groups in terms of other species or functional genes [19]. In other studies, *Prevotella* and *Leprotrichia* genera were noted to be significantly higher in children with cavities at 6.5 years of age [18]. Aside from these groups, *Lactobacillus*, *Actinomyces*, and *Propionibacterium* are also thought to have a role in the formation and progression of caries [20,21]. Importantly, despite some inaccuracies between reports, it is more widely recognized that significant shifts in oral microbiome occur not only during caries development but also in the preclinical phase, offering the possibility to utilize this phenomenon as a biomarker for early diagnosis and prediction of caries [22].

Periodontitis is a prevalent bacterial disease that poses a risk for some systemic illnesses, including cardiovascular diseases [23], diabetes [24], and cancer [25]. Due to their proximity to the gingival vasculature, the periodontal pockets, which contain potentially inflammatory periodontal flora, can be disseminated to distal anatomical sites [26]. In a recent study, 10 genera, including *Moraxella*, *Fretibacterium*, and *Treponema*, were noted as predominant in periodontitis, justifying the screening of periodontitis through an analysis of the composition of the oral microbiota [27]. Other studies demonstrate the significance of *P. gingivalis*, *Bacteroides forsythus*, and *Treponema denticola* for the development of this pathology and justify bacteria-targeting treatment of periodontitis using antimicrobials [28]. Specifically, *P. gingivalis* was found to stimulate macrophages, leading to the creation of an inflammatory milieu defined by the production of cytokines (TNF-α, IL-1β, IL-6), prostaglandins, and metalloproteinases (MMPs), thereby contributing to tissue destruction, which is characteristic of periodontitis. Differences in the oral microbiome were also detected for individuals with oral implants developing peri-implantitis [29]. Several mucosal diseases (e.g., oral leukoplakia, lichen planus) and some oral cancers have also been connected to the imbalance of the oral microbiota [30]. Moreover, the microbiota also has complex interactions with different viral particles, affecting them directly or indirectly [31]. There are also hypotheses suggesting the protective role of microbiota in, for example, rotavirus or influenza infection by different mechanisms [32,33].

## 3. Expression of Endogenous Defense Peptides in the Oral Cavity and Their Role in Maintaining the Balance of the Oral Microbiota

Due to the abundance of bacteria, both beneficial and harmful, in the oral cavity, maintaining a delicate balance within the microbiota requires multiple responsive and efficient defense systems to be produced by the host. The oral epithelium is a crucial element in this process since it acts as a physical barrier against infection and also actively contributes to innate host defense. Epithelial cells play a vital role in responding to bacterial exposure through supra- and subgingival biofilms on the tooth surface or bacteria attached to mucosal surfaces by secreting chemokines and cytokines to attract neutrophils. Importantly, they also produce natural AMPs and proteins in a continuous process and as a reaction to bacterial interactions. Further studies on the presence of AMPs in the oral environment revealed that AMPs are released by nearly all cells and tissues present in the oral cavity, i.e., tongue [34], palatine tonsils [35], salivary glands [36], sulcular epithelium [37], junctional epithelium [37], and neutrophils [37,38]. AMPs were noted to also be transcribed and expressed in pulp tissues [39]. Over 2000 proteins are detected in saliva [40,41], and over 100 in the gingival crevicular fluid [42], highlighting their relevance for maintaining oral environment health. Among a spectrum of beneficial functions of AMPs in the oral cavity, antimicrobial [43], biofilm disrupting [44], immunomodulatory [45], regenerative [46], and microbiota-modulating features [47] are the most recognized and accepted. Nevertheless, the link between AMP expression and cancer development also requires consideration [48].

Among the AMP group, defensins, cathelicidins, and histatins are accepted as crucial in the human mouth [49], although other AMPs such as calprotectin and adrenomedullin should also be recognized [50]. As there is a substantial body of literature concerning these antimicrobial properties in the oral cavity, including their structures and functions [11,51,52], in this review, we focus on the most recent reports, describing novel discoveries and achievements in the field of the physiological functions of AMPs, alterations in their expression in the course of various medical conditions, particularly those of the oral cavity, and the utility of AMPs for the prognosis, diagnosis, and treatment of diseases in the oral environment.

Recently published reports expanded our knowledge of the expression of AMPs under different pathological conditions. Most recently, the beta-defensin index (BDI), which is calculated as a ratio of human β-defensin-3 (hBD-3) to human β-defensin-2 (hBD-2), was presented as a viable and economical method for detecting oral squamous cell carcinoma (OSCC) [53]. Such a statement was made based on the previously published data revealing pro-tumorigenic activities of hBD-3 compared to hBD-2 [54]. In another study, Zhang et al. demonstrated that not only are hBDs downregulated in periodontitis, but they are also correlated with lower immune infiltration and are regulated by vitamin D_3_ via CYP27A1 in human gingival fibroblasts [55]. Previous studies demonstrated alterations in AMP expression in periodontitis [56,57], including deficiencies associated with environmental factors, including smoking [57]. The relevance of vitamin D in dental health was also highlighted recently using an ex vivo model of dental caries. Nireeksha et al. confirmed that salivary vitamin D statistically decreased with increasing severity of caries, indicating that the stimulation of AMP LL-37 production by vitamin D is crucial for the prevention of caries [58]. Following other studies [59], Rani et al. confirmed the relevance of human neutrophil peptide (HNP1-3) for the prevention of caries in children [60]. Notably, an interesting approach to maintaining antimicrobial effects in the oral environment was also recently demonstrated by Nittayananta et al. The authors developed a curcumin-containing spray that displayed antiviral activities against SARS-CoV-2 and influenza viruses, and confirmed that application of the spray inhibited viruses while maintaining cell viability via a mechanism involving augmentation of LL-37, human defensin-5, and cytokine expression, thus enhancing mucosal innate immunity [61].

Studies carried out in the last 2 years also revealed novel molecular mechanisms of action and biological characteristics for well-recognized oral AMPs. Campbell et al. revealed that histatin-5 (Hist-5), an AMP found in human saliva, exerts its fungicidal activity by facilitating the hyperaccumulation of copper in *C. albicans* cells [62]. Concurrently, a study conducted by Stewart et al. strongly recommended reassessing the role of Zn(II) in histatin-5 function and the role of this peptide as a salivary antimicrobial agent; under in vitro conditions, histatin-5 does not contribute to Zn(II)-dependent nutritional immunity against streptococci but instead suppresses the availability of excess zinc ions to bacterial cells in vitro, potentially reducing the virulence of bacteria in the oral cavity and oropharynx [63]. Considerable emphasis has also been placed on human cathelicidin LL-37, a widely acknowledged host AMP in the oral cavity, which has been previously detected in saliva and gingival crevicular fluid of patients with chronic forms of periodontitis and gingivitis at substantially elevated concentrations [64]. However, the antibacterial properties of this molecule and its ability to regulate the activity of inflammatory cells in the oral cavity have not been well investigated until recently. Lappin et al. showed that LL-37 not only has antimicrobial properties but also directly inhibits the expression of inflammatory cytokines induced by lipopolysaccharides from *E. coli* and *Porphyromonas gingivalis*. Additionally, it promotes the expression of various bioactive molecules involved in inflammation and repair, such as CXCL8, hepatocyte growth factor, and CXCL1, suggesting that LL-37 or cathelicidin-derived mimetics could be used as a therapeutic option for treating periodontitis [65].

Recently published studies highlight the possibility of improving the diagnosis of systemic medical conditions via analysis of salivary markers, including AMP concentrations. Analysis of salivary proteome was proposed recently as a method to diagnose Parkinson’s disease (PD) and distinguish it from similar pathologies, including Alzheimer’s disease (AD). The saliva of PD subjects showed a higher abundance of antileukoproteinase (or secretory leukocyte proteinase inhibitor) and cystatin SA, while the saliva of AD patients showed the highest levels of α-defensins and short oxidized S100A9 peptide [66]. Other studies observed a lowered expression of AMPs, including human cathelicidin LL-37, in children and adolescent subjects living with HIV following antiretroviral therapy [67]. Disrupted levels of AMPs from lysozyme, lactoferrin, or cathelicidin were also noted in subjects upon SARS-CoV-2 infection [68,69]. Likewise, abnormalities in the salivary profiles of defensins and thymosin β4 have been found in West and Noonan patients with developmental non-inflammatory gingival enlargements [70].

Although the most clinically relevant therapeutic approaches still rely on antimicrobial agents, there is an ever-growing recognition that dental treatment strategies that seek to restore oral cavity balance are superior to those that eliminate a specific pathological species [71]. In this aspect, Zeng et al. proposed the utility of three *Lactobacilli* strains for preventing *S. mutans* and *C. albicans* growth in models imitating clinical cariogenic conditions and demonstrated that *L. plantarum* 14917 and the AMP produced by this organism—plantaricid—had an inhibitory effect on the expression of *S. mutans* and *C. albicans* virulence genes, as well as on the formation of *C. albicans* hyphae [72]. As an agent to improve oral health, microcapsules containing the probiotic *Streptococcus salivarius* LAB813 strain were developed by Choudhary et al. [73]. A similar approach was tested most recently by Nogueira and co-workers who developed a chewing gum containing *Lactobacillus* spp. and *Bifidobacterium* spp. in both lyophilized and microencapsulated form. In an in vitro investigation, they found that both microorganisms had a strong capability to limit the growth of cariogenic *Streptococcus mutans* UA 159, and this was attributed to the generation of AMPs, which caused the cariogenic bacteria to be inhibited. Significantly, the inhibitory effects of this method were similar to those of the oral antiseptic chlorhexidine 0.2% under laboratory conditions, indicating its strong potential for clinical use [74].

## 4. Natural AMPs, Their Synthetic Analogs, and Their Potential Utility in the Treatment of Oral Diseases—Recent Insights

Due to the spectrum of beneficial features of AMPs, including antimicrobial [43], immunomodulatory [45], and regenerative [46] activities, AMPs have been evaluated as therapeutic agents for the treatment of oral diseases such as halitosis [75], caries [76], mucositis [77], and periodontitis [78]. Several recent reviews [43,52,79] describe the therapeutic potential of defensins against bacterial, fungal, and viral pathogens. Moreover, a compelling amount of data suggest their utility in the diagnosis and treatment of head and neck cancers [80]. Attention has also turned to milk AMPs. Through the utilization of an integrated approach that incorporates in vitro proteolysis optimization, antimicrobial activity assays, proteomics profiling, and MBPDB database matching, researchers have successfully isolated and proven the efficacy of casein-derived peptides against oral pathogens, specifically *S. mutans* and *P. gingivalis*, in both planktonic and biofilm states [81]. Although the activity of the milk-derived peptides themselves did not differ significantly from other peptides of natural origin, the authors’ high-throughput approach itself directed further research on other peptides for oral disease therapy.

In addition to highly promising results obtained using naturally occurring AMPs, recent studies have also involved synthetic AMPs, developed based on both human and non-human sources. Such an approach is highly understandable and justified considering some crucial limitations for naturally occurring antimicrobials, including hampered extraction and problems with large-scale preparation, short half-life, susceptibility to protease degradation, inactivation by physiological salt concentration, and the presence of saliva [82]. For these reasons, a spectrum of modifications to AMPs has been made, namely polymerization of α-amino acid N-carboxyanhydride, PEGylation, usage of D-amino acids, or unnatural amino acids, cyclization, lipidation, and backbone modifications [83]. One such modified peptide is [W^7^]KR12-KAEK, which was developed by the substitution of isoleucine to tryptophan at the seventh position and the addition of KAEK to the sequence of KR-12 peptide (i.e., 18–29 amino residues of LL-37). As Vasconcelos et al. demonstrated, [W^7^]KR12-KAEK exhibited potent anti-*E. faecalis* activity in contrast to the inefficient KR-12 peptide, and they concluded that this difference in peptide activity resulted in interactions of specific amino acids within the sequence. Specifically, the substitution for tryptophan, which is prevalent in many AMPs due to its hydrophobicity and charge, as well as the insertion of the KAEK sequence at the C-terminal position of KR-12, caused an increase in the peptide chain size and altered its charge and amphipathicity [84]. A favorable effect of peptide modification was also demonstrated for DJK-5, a ᴅ-enantiomeric cationic peptide, acting via targeting intracellular ppGpp accumulation and thus, modulating the stress response in bacteria [85]. In previous studies, DJK-5 attracted extensive attention for its broad-spectrum antibiofilm capability [86]. Accordingly, Hu et al. compared its antibiofilm activities with commercial mouthwashes using a model of dental plaque-derived biofilm grown on the surface of dental restorative materials and demonstrated that DJK-5 outperformed conventional mouthwashes in killing bacteria in oral multispecies biofilms [87]. Among the other most recently described antimicrobials, particular attention was given to lactotransferrin-derived AMP LF-1, which is based on the N-terminus of lactotransferrin. Luo et al. demonstrated that LF-1 and LF-2 peptides were effective against *S. mutans* and *Actinomyces* species, both in planktonic and biofilm forms, while displaying satisfactory biocompatibility in a rodent model of dental caries [88]. Further studies demonstrated strain selectivity of the peptide, confirming that LF-1 targeted *preferably S. mutans* over oral commensal bacteria, including *Streptococcus sanguinis* and *S. gordonii* [89]. Most recently, a considerable gap in the knowledge of the mechanisms of the antimicrobial activity of this molecule has been filled. It was demonstrated that LF-1 peptides limit the acidogenicity and aciduricity of *S. mutans*, and inhibited the adhesion and EPS (exopolysaccharide) synthesis of *S. mutans*, resulting in impaired biofilm formation. Moreover, LF-1-mediated the downregulation of *S. mutans* gene expression related to cariogenic virulence, and the stress response [90] demonstrated the benefit of this molecule in a potential dental caries treatment. An innovative approach to developing novel AMP-based antimicrobials was also demonstrated by Zhang et al. As described recently, the authors synthesized a novel synthetic peptide GAPI via grafting AMP polyphemusin I (PI; from the American horseshoe crab *Limulus polyphemus*) to gallic acid (GA), which also displays antimicrobial properties [91]. A conjugate with broad-spectrum activity against oral microbes, including *L. rhamnosus*, *L. acidophillus*, *Actinomyces naeslundii*, *E. faecalis*, and *P. gingivalis*, was generated, although no selective activities among strains were noted [91]. The possibility of modulating the oral microbiome using AMP-derived synthetic peptides was demonstrated for other derivatives. The LL-31 peptide, a truncated variant of LL-37 lacking the 6 C-terminus amino acid residues as well as D-LL-31, was found to modulate the composition of saliva-derived microbial biofilm via decreasing the relative abundance of *Campylobacter* while increasing the abundance of *Lactobacillales* simultaneously. In *P. gingivalis*-enriched biofilm models, the efficacy of the peptide function was impaired, indicating the significance of dysbiosis states for the activity of AMPs [92]. AMP-induced modulation of the oral microbiome was also observed in another clinical study [93] (Table 1). In addition to these reports, new data were recently presented on oral bacteria-produced lantibiotics and their potential use in the treatment of dental disorders [94]. Currently, there is a significant increase in the interest in lantibiotics as alternatives to conventional antibiotics. Barbour et al. demonstrated an unidentified structural class of three related lantibiotics (named collectively salivaricin 10), produced by *Streptococcus salivarius* and reported their targeted antimicrobial properties against known oral pathogens, including multidrug-resistant organisms as well as multispecies biofilms. Importantly, regardless of high antibacterial efficiency against *Streptococcus pneumoniae*, vancomycin-resistant *Enterococcus faecium*, *P. gingivalis*, or *Neisseria gonorrhoeae*, salivaricin 10 did not inhibit *S. mutans* and *L. rhamnosus*, demonstrating a considerable selectivity toward pathogenic bacteria. Importantly, phosphorylation of salivaricin 10 was demonstrated as determining the unreported immunomodulatory effects, including enhancing neutrophil recruitment and phagocytosis and promoting anti-inflammatory macrophages. As such, further development of salivaricin 10 as a multifaceted molecule with antimicrobial, antibiofilm, microbiome-regulating, and inflammatory-resolving properties is needed [94].

Research in recent years has also focused on the development of new pharmaceutical formulations that allow the beneficial properties of already-established AMPs to be used to treat local and generalized oral conditions. Most recently, a randomized controlled clinical trial was conducted to evaluate the antibacterial effectiveness of AMP-based mouthwash against oral pathogens and elucidate its efficacy in reducing halitosis and supragingival plaque. More detailed BOP^®^ mouthwash consisting of domiphen (antimicrobial with cationic surfactant property and membrane-permeabilizing characteristics [99]) and two AMPs with antibacterial, anti-inflammatory, and immunomodulatory activities, namely ε-PL (ε-poly-L-lysine) and FP (funme peptide [100]), was demonstrated to display bactericidal effects against *F. nucleatum*, *P. gingivalis*, *S. mutans*, and *Aggregatibacter actinomycetemcomitans* and exhibit anti-dental plaque and anti-halitosis properties in a clinical trial while maintaining a satisfactory level of biocompatibility [75]. Additionally, a few other formulations were also explored in clinical trials in the last 5 years, with some results recently published (Table 1). Favorable outcomes recorded in these studies strongly confirm the clinical utility of AMP-containing pharmaceutics. Clinical trials of some other AMP-containing pharmaceutics designed to improve the prevention and treatment of periodontal disease and dental caries are also listed in Table 1.

To improve the therapeutic utility of AMPs in periodontal disease treatment, Amer et al. constructed two enzyme-activatable prodrugs by combining P-113 AMP (i.e., the 12-amino-acid derivative of Histatin-5) with a charge-shielding toxicity-quencher and a recognition sequence for SAP9 (secreted aspartyl proteases) or RgpB (gingipains) for efficient detection and targeting of *C. albicans* and *P. gingivalis*, respectively [101]. By offering the targeted release, the prodrug peptides demonstrated high antimicrobial effects toward the microorganisms expressing the trigger protease without considerable toxicity when targets were absent [101]. Histatin-5 was also used as a base for the production of mucoadhesive gel for the treatment of oral mucositis [77]. Research performed both in vitro and in vivo confirmed that a Hist-5/Carbopol 934/hydroxypropyl methylcellulose-containing formulation gave a sustained release profile of Hist-5, antimicrobial activity against *S. aureus*, *P. aeruginosa*, *E. coli*, and *C. albicans*, as well as wound healing-promoting capabilities, justifying further development and utilization of such formulations [77].

Significant progress was also achieved in the development of enhanced biomaterials incorporating AMPs. Most recently, Dai et al. presented a new AMP-containing scaffold showing the potential for treating bone abnormalities, particularly in the craniofacial region. As such, GL13K (i.e., AMP derived from protein saliva) was incorporated into a collagen membrane by self-assembly to create a new bone regeneration scaffold to reduce post-surgical infections and aid in bone regeneration in dental and orthopedic applications. The authors confirmed that a synthetic, self-assembling nanofibrillar membrane displayed both antimicrobial activities and enhanced bone formation in vivo, expanding the knowledge on the utility of AMPs for the fabrication of improved, dual-action oral biomaterials [102]. In another study, Zhou et al. aimed to improve the effects and characteristics of microcin C7 (McC7) for use in the treatment of periodontitis. McC7 [78] is an aspartyl tRNA synthetase “Trojan horse” inhibitor [103] and is characterized by low protease resistance and susceptibility to unique oral environments such as mastication, salivary flow, and tongue movement. An injectable temperature-sensitive sustained-release hydrogel was proposed as the base for a drug delivery system of this material. Results demonstrated the slow and continuous release of McC7 with simultaneous good biocompatibility and biodegradability accompanied further by antibacterial and anti-inflammatory effects in a periodontitis rat model, which indicated the validity of using such systems in dental diseases.

Achievements of nanotechnology have also been utilized for the fabrication of next-generation antibacterial materials and formulations. Polymeric nanopolymers have recently gained attention for use in the treatment of chronic oral inflammatory diseases of bacterial origin, periodontitis in particular [104]. Using another approach, to improve the therapy against multispecies biofilms related to periodontitis, de Carvalho et al. proposed conjugating blue light-activated chlorin-e6 (Ce6) with LL-37 in the form of a nanoemulsion for photodynamic eradication of oral pathogens. The combination of a photosensitizer (PS) with an AMP which permeabilizes bacterial membranes and, thus, permits the penetration of PS and its subsequent intracellular effects was demonstrated as highly efficient. The development of a nanoemulsion concurrently achieved improvements in substance stability, greater surface area for enhanced cellular administration, and an enhanced bioavailability and biodistribution of molecules [105]. Silver nanoparticles with the self-assembling GL13K peptide were also used as a nanocoating material for enhanced osseointegration in titanium mini implants [106]. The DK5 peptide was also evaluated, which was derived from histatin-1 and further encapsulated into a liposomal delivery system (DK5-Lips) for remineralization of initial enamel caries. Researchers confirmed both in vitro and in vivo that DK5-Lips displayed a favorable slow-release effect of the peptides. Simultaneously, such developed nanoliposomes also demonstrated good stability in saliva, while promoting a higher amount of remineralization and reducing the extent of tooth decay [76].

Significantly fewer recent literature reports have addressed the use of natural AMPs and their synthetic analogs in treating head and neck cancers, including oral cancers. Among those presented, piscidin-1, an AMP derived from the mast cells of hybrid striped bass (*Morone saxatilis* × *M. chrysops*), in addition to its antibacterial properties, induces ROS-mediated apoptosis in oral squamous cell cancer cells and inhibits the angiogenesis, demonstrating the ability to limit cancer spreading by both direct and indirect mechanisms [107,108].

A unique advantage in the treatment of periodontal diseases has recently been attributed to the development of nanofibrous membranes with antibacterial, anti-inflammatory, and tissue regenerative properties, prepared by electrospinning. To date, a compelling number of reports demonstrate the great clinical potential of electrospinning-prepared biomaterials for wound dressings [109], cancer treatment [110], and tissue engineering [111]. The extensive range of medical applications, including dentistry, is attributable to the distinctive properties of these biomaterials, which include (i) their capacity to replicate the scale and morphology of extracellular matrix (ECM) proteins, thereby promoting cell attachment, proliferation, and differentiation, (ii) high porosity that permits oxygen permeation, and (iii) the ability to enhance drug loading and ensure localized, sustained, and controlled drug delivery [112,113]. Although a majority of reports on the antimicrobial properties of electrospun biomaterials engage conventional antibiotics [114], polymers [115], or nanoparticles [116] as factors limiting the spreading of infection, there are also a few studies demonstrating that such prepared biomaterials might be incorporated with AMPs. In one report, LL-37 and plant-derived polyphenol were used to modify nanofibrous scaffolds to obtain a material with antimicrobial features, anti-inflammatory effects, and the ability to promote bone regeneration [117]. Similarly, lysozyme was integrated into a mucoadhesive electrospun patch, creating a drug delivery system with significant promise for administering therapeutic proteins to the oral mucosa [118]. In another study, Pac-525, a short, tryptophan-rich AMP with antimicrobial activity against *S. sanguis*, *F. nucleatum*, and *P. gingivalis*, was released over extended periods from nanofibrous membranes, inhibiting bacterial infections for up to one month while accelerating bone regeneration [119]. These studies encourage continued research on the creation of AMP-containing electrospun biomaterials, despite the numerous hurdles that remain in attaining clinical treatment for patients.

Collectively, cited reports suggest that AMPs have significant application potential in the treatment of oral disorders, which is related to their multidirectional antimicrobial activity, including not only direct lethal effects but also indirect effects such as the induction of phagocytosis processes. Thus, further research into the modification of AMPs to improve their stability, biological activity, or biocompatibility is justified and greatly needed.

## 5. Mimicking AMPs as an Approach to Improve Oral Health Maintenance: Ceragenins—Lipid Analogs of AMPs

As stated above, the primary way to modulate the biological activity of natural AMPs, both in terms of biological activity and their selectivity to bacterial cells or toxicity to host cells, is to alter the amino acid sequence to change its basic physicochemical characteristics, such as charge or hydrophobicity. Ultimately, such changes lead to the modulation of the peptide’s internalization into the biological membranes of the pathogen, allowing for the exertion of a direct antimicrobial effect. On the other hand, mimicking the specific characteristics of AMPs that determine their membrane-interacting abilities, in particular, positive charge and facial amphipathicity, is an innovative way to obtain new biologically active molecules. Using such an approach, ceragenins were developed as non-peptide mimics of AMPs [120]. Ceragenins are derived from cholic acid, a common bile acid that inherently displays facial amphipathicity and is secreted into the gastrointestinal tract to aid in the solubilization of lipids. Because they are based on cholic acid, ceragenins were originally referred to as cationic steroid antibiotics, but to eliminate any implication that these compounds could have steroid-like characteristics, they were renamed as ceragenins. Importantly, since ceragenins are based on the structure of cholic acid, they are not substrates for proteases [121], which are ubiquitous physiologically and are commonly produced by pathogenic microorganisms [122]. Ceragenins are well tolerated by local administration [123]. A key characteristic of ceragenins is that their preparation is simpler and less expensive than the peptides they mimic [124,125] (Table 2).

Collectively, ceragenins are highly efficient against Gram-positive and Gram-negative bacteria [126], both aerobic [127] and anaerobic [124], as well as against yeast and filamentous fungi [128], and viral pathogens [129]. Such broad-spectrum activity of ceragenins is proposed to be due to their ability to associate with microbial and viral membrane components and induce membrane disruption, likely by the carpet model mechanism. Studies performed by Ding et al. demonstrated that the target of ceragenins with Gram-negative bacteria is the lipid A portion of lipopolysaccharide (LPS), which assures a considerable selectivity of these compounds toward bacterial cells over the host cells [130]. Interestingly, in Gram-positive bacteria, which do not produce LPS, the bactericidal activity of ceragenins was correlated with a content of phosphatidylethanolamine in the bacterial membrane [131]. With either type of target, the ability of ceragenins to depolarize and permeabilize microbial membranes is recognized as crucial for their killing activities [132]. Some studies also describe the ceragenin-mediated induction of oxidative stress due to the overproduction of reactive oxygen species in treated microbial cells [133]. In addition to this membrane-based-established mechanism of activity, most recent research carried out using a combination of whole-genome sequencing (WGS) and transcriptome sequencing (RNA-seq) revealed that ceragenin CSA-13 promotes the downregulation of acid stress-associated genes and a distinct dysregulation of genes involved in carbon metabolism, resulting in a switch from pyruvate fermentation to the glyoxylate pathway [134]. As such, ceragenins as metabolism-regulating molecules should also be recognized apart from their membrane-permeabilizing features. Moreover, in addition to the potent activities of ceragenins against planktonic microorganisms, they also display considerable antibiofilm activities, which are associated with both inhibiting the formation of microbial—both single and dual species—biofilms and disrupting those already established. Most recently published studies demonstrated that ceragenins, apart from their inhibiting abilities against biofilm, actively alter the mechanical properties of these bacterial communities by increasing their fluidity, and thus, making them more prone to the killing action of antimicrobials and easier to eradicate mechanically [135]. Such abilities are highly beneficial in the prevention and treatment of biofilm-associated infections, including those spreading in the oral environment.

**Figure 1:**
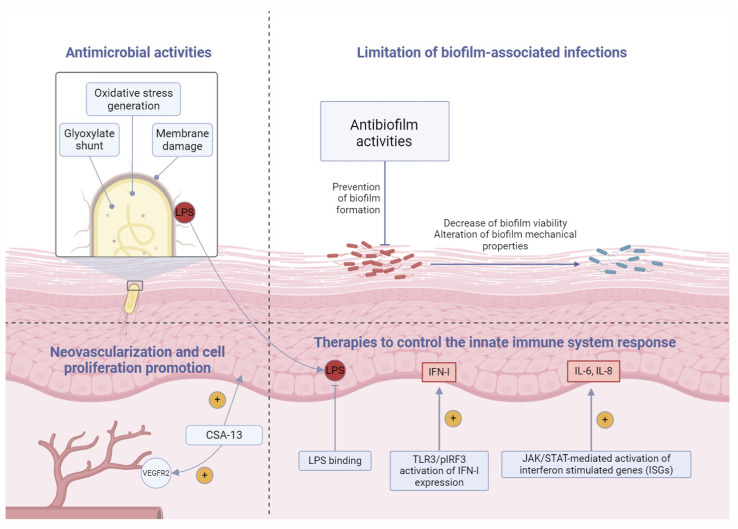
The biological activities of ceragenins. Details on the molecular mechanisms of reported effects are described in more detail below.

It is crucial to acknowledge that ceragenins, in addition to their antimicrobial properties, exhibit a range of non-antimicrobial actions analogous to other AMPs, including regenerative, anti-inflammatory, and immunomodulatory effects (Figure 1).

Although the data on regenerative properties of ceragenins are limited, reports demonstrate their ability to stimulate the proliferation of human keratinocytes [136] and human osteoblasts [137] at concentrations that are non-hemolytic and bactericidal. Moreover, one study performed by Olekson et al. [136] demonstrates that ceragenins promote tube formation via a VEGFR2-dependent mechanism increasing their regenerative potential. The ability of ceragenins to sequester LPS and neutralize its cellular effects was confirmed by Bucki et al.; as demonstrated, the preincubation of LPS with CSA-13 resulted in a loss of pro-inflammatory activities of LPS to endothelial cells, and this effect was comparable to the activity of the naturally occurring human LL-37 peptide [138]. In another study, immunomodulatory activities of ceragenins were demonstrated; Suprewicz et al. showed that ceragenins exhibit antiviral activities, which are attributed to the stimulation of antiviral cytokines, such as type I interferons via TLR3/pIRF3 signaling pathway. Concurrently, the binding of IFN I to its receptor (IFNAR) activates the downstream JAK/STAT pathway, leading to the generation of interferon-stimulated genes (ISGs) such as IL-6 and IL-8 [139]. Although those data were obtained in COVID-19-mimicking experimental settings, they highlight the potential beneficial effects of ceragenins in the prevention and treatment of other inflammatory-related medical conditions.

## 6. The Breadth of the Spectrum of Antimicrobial Activities of Ceragenins and the Possibility of Their Use in the Treatment of Oral Diseases

Over the last few years, ceragenins were thoroughly tested against a spectrum of bacterial, fungal, and viral pathogens, demonstrating a promising class of cationic lipid antibiotics. Among the spectrum of microorganisms against which ceragenins have shown high bactericidal activity is a wide range of oral pathogens, including cariogenic and periodontitis-associated organisms. However, much of this characterization was performed using experimental conditions distinct from the oral environment. It is important to note that the clinical potential of these compounds has been considerably enhanced by the observation of activity against a significant number of these microorganisms in both planktonic cells and the biofilm they form. Table 3 summarizes the reports on the antimicrobial activities of ceragenins against microorganisms associated with oral cavity infections.

Among these studies, research demonstrated by Isogai et al. deserves particular attention. The authors explored the antimicrobial activities of CSA-13 against *S. mutans* (n = 24), *P. gingivalis* (n = 12), *P. candigingivalis* (n = 2), and *P. circumdentaria* (n = 10) and established MIC values of ceragenins against strains ranging from 1 to 16 µg/mL, demonstrating a great potential of this molecule in oral cavity infections. Notably, the activity of CSA-13 was significantly greater than the 27 amino acid domain of CAP18/LL37 (hCAP18_109-135_) and BMAP-28 peptide. For all tested isolates, minimal bactericidal concentrations (MBCs) did not exceed 16 µg/mL [144], which is well below the hemolytic concentration of this ceragenin [140]. In another study, Leszczyńska et al. established the broad spectrum of ceragenins CSA-13, CSA-90, and CSA-92 as antimicrobials against pathogens associated with oral and upper respiratory tract infections, including *S. mutans*, *S. pyogenes* or *E. faecalis*. Following the previous report, all tested ceragenins were highly efficient against planktonic bacterial strains, with MIC values ranging from 0.35 to 8 µg/mL, and this effect was at lower concentrations than those required for the activity of the endogenously produced human cathelicidin LL-37 [145].

One of the most important advances in terms of the significance of ceragenins in combating oral pathogens was achieved by Durnaś et al. In one of their papers, the authors demonstrated that ceragenins CSA-13 and CSA-131, as well as iron oxide-based nanosystems containing them, showed significant antimicrobial activity against a range of anaerobic microorganisms present in the oral cavity with ceragenins’ MIC values in the range of 0.5–16 µg/mL. Although the authors did not evaluate the therapeutic potential of ceragenin strictly in terms of oral infections and only two clinical strains used in the study (*Prevotella oralis* and *P. melaninogenica*) were isolated from postoperative wound in the oral cavity region, the research conducted indeed confirmed the significant potential of these molecules in the eradication of bacteria from *Bacteroides*, *Clostridiales*, and *Peptostreptoccus* genera [124]. Other research demonstrated comparable activity, including against *Clostridioides difficile* [146]. A considerable number of studies have also been published in the aspect of fungicidal activities of ceragenins. As oral candidiasis is considered as one of the most frequent oral infections, particularly in immunocompromised subjects, both prophylactic and therapeutic approaches are required to ensure the oral safety of this group of patients. In such aspects, ceragenins could be highly beneficial as several reports demonstrate their effectiveness against *Candida albicans* and *C. glabrata* fungi, which are recognized as most clinically important for proper oral hygiene. Multiple types of ceragenins demonstrated activity against both *C. albicans* and non—*C. albicans* isolates, including first- and second-generation ceragenins CSA-8, CSA-13, CSA-44, CSA-131, CSA-138, CSA-142, and CSA-192. As demonstrated in a few studies, MIC values of these ceragenins against many laboratory and clinical strains were as low as 0.25 µg/mL, with activity comparable to or better than clinically used antifungals agents, including fluconazole and amphotericin B [128,140,141,142].

Ceragenins are effective against microbes that are less recognized as oral disease-causing pathogens but are highly associated with the development of systemic medical conditions arising from oral dysbiosis. *H. pylori* is mostly found on the gastric mucosa in humans and is closely linked to the development of gastritis. However, epidemiological research has shown that individuals with dental caries or inadequate oral hygiene also carry *H. pylori* in their oral cavity [147]. A recent literature review has shown a strong link between the presence of *H. pylori* infection in the mouth and an increased occurrence of dental caries in patients, even in the absence of gastric infection. This suggests that the oral cavity serves as another environment for *H. pylori* and may be the origin of infection, re-infection, and transmission to the stomach [148]. Thus, maintenance of an appropriate oral microbiota composition might not only limit dental caries development but also prevent gastrointestinal complications. In their two independent studies, Leszczyńska et al. demonstrated that ceragenins, including CSA-90 and CSA-92, but mostly CSA-13, display antibacterial effects against *H. pylori*, both laboratory and clinical isolates. Although the initial research was focused on the activity of CSA-13 against *H. pylori* in simulated gastric juice (i.e., low pH and in the presence of pepsin), it confirmed that CSA-13 resisted proteolytic degradation and inhibition by mucin [149]. In the second study [143], the authors demonstrated that the antibacterial activity of ceragenins is retained in the presence of saliva and dental plaque, strongly suggesting the clinical applicability of ceragenins in the eradication of oral pathogens. The potential for the clinical use of ceragenins is also strengthened by reports demonstrating the potent killing activity of these compounds against *S. pneumoniae*, bacteria that often colonize the oropharynx and can lead to community-acquired pneumonia. In several independent studies, ceragenins CSA-13, CSA-44, CSA-90, CSA-92, and CSA-131 were highly efficient against this pathogen both in planktonic and biofilm forms [150]. Likewise, *S. pyogenes* was also susceptible to low concentrations of ceragenins (MIC values against a clinical isolate of *S. pyogenes* ranged from 0.7 mg/L for CSA-13 and CSA-92 to 1.4 mg/L for CSA-90) [143].

**Figure 2:**
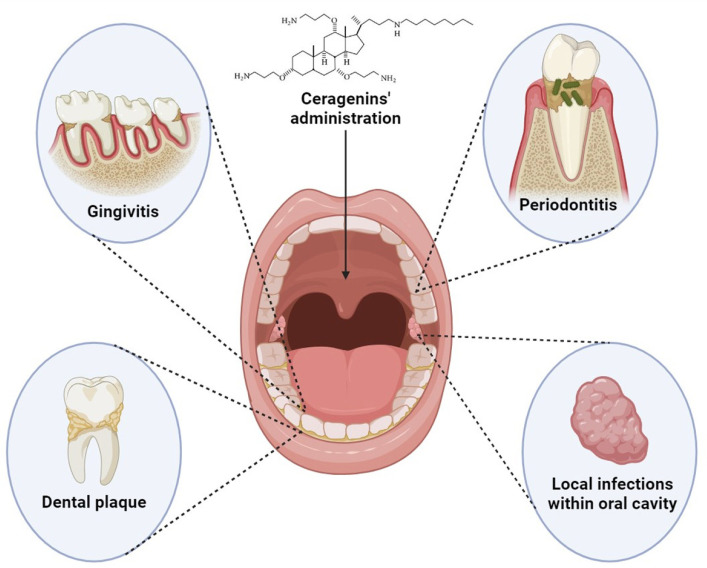
Potential applications of ceragenins in the treatment of oral diseases.

In contrast to pathogenic microbes, the bactericidal activity of ceragenins against *Lactobacillus* spp. was considerably lower (MIC range: 22.4–46.8 µg/mL), pointing out the limited susceptibility of commensal strains of bacteria to host antibacterial agents when compared to pathogenic ones [143]. Such selectivity may contribute to the microbiota-modulating capabilities of ceragenins, which would be highly beneficial for dysbiosis-associated oral diseases, including periodontitis [143]. Nevertheless, there are a limited number of studies aimed at elucidating such biological features of ceragenins. In one report, Wang et al. showed that the administration of CSA-13 combined with Eudragit and methylcellulose (an oral formulation that releases CSA-13 gradually in the terminal ileum and colon, where the pH is alkaline) to mice infected with *C. difficile* has a beneficial effect on the microbial composition of the intestinal tract. In this study, CSA-13 decreased the abundance of *C. difficile* in fecal samples of mice, while simultaneously increasing the abundance of *Peptostreptococcaceae* bacteria, *Akkermansia*, and *Lactobacillus* genera [146]. This suggests that CSA-13 restores the balance in the intestinal microbiota during infection. Conversely, CSA-44 was shown to not influence the structure of the cecal microbiota in broiler chicks possessing a low or high diversity of enteric microbiota, but instead, it altered the function of enteric bacterial communities as revealed by metabolomic analyses [151]. To date, no research aimed at investigating the microbiota-modulating capabilities of ceragenins in oral environment settings has been published, and this topic surely requires further exploration. At the same time, a broad spectrum of pathogens against which ceragenins exert antimicrobial effects highlights their clinical potential as agents against infections and infection-associated diseases in the oral cavity environment (Figure 2).

## 7. Activity of Ceragenins in the Presence of Saliva and Dental Plaque

To ensure the clinical applicability of ceragenins for the prevention and therapy of oral cavity disorders, it is pivotal to ensure that their killing activities are retained in the presence of fluids in oral environments, including saliva or dental plaque. Saliva, as is widely acknowledged, is an essential element of the oral environment. It not only ensures proper digestion, moisturizes the mouth, neutralizes toxic acids, and promotes the remineralization of teeth, but also maintains oral health through the presence of a variety of AMPs [152]. It is well recognized that although pathogens generate an extracellular matrix by utilizing endogenous constituents in saliva during the biofilm development process [153], the antimicrobial effects of AMPs restrict the excessive growth of microbial populations. Simultaneously, components of saliva such as mucin or proteases limit the effectiveness of some endogenous antimicrobials, such as human cathelicidin LL-37 [154] or exogenous disinfectants (e.g., chlorhexidine [155,156]). Compelling evidence demonstrates that ceragenins, due to their non-peptide chemical structure, are resistant to proteases, which are present in the oral cavity, as well as retain their antimicrobial activity in the presence of human saliva. To date, a few studies demonstrated that ceragenins, both in free form and attached to the surface of metallic nanoparticles, display potent bactericidal and fungicidal activity in the presence of human saliva.

Bucki et al. showed that, in contrast to human cathelicidin LL-37, which is partially inactivated by salivary mucins, ceragenin CSA-13 displays potent antimicrobial effects against *P. aeruginosa* and *E.coli* both in the presence of purified mucins or whole saliva, suggesting its possible application in the treatment of oral infection [154]. This effect was confirmed in two other experiments by Leszczyńska et al.; in one of the studies, CSA-13 demonstrated its activity against *P. aeruginosa* [157], while in the other one—against *S. salivarius*, *S. mutans*, *E. faecalis*, and *H. pylori* [143]. Notably, the maintenance of antimicrobial activity of ceragenins in the presence of saliva was also confirmed with *Candida* fungi. As demonstrated by Niemirowicz et al. [137] as well as Tokajuk et al. [158], ceragenins CSA-13 and CSA-44 limit the viability and biofilm formation by yeast (or yeast in mixture culture with *E. faecalis*) upon the addition of saliva (50%). Likewise, the potent antimicrobial activity of ceragenins was also maintained in the presence of dental plaque, as a tooth-borne complex biofilm composed of a broad and varied microbial community (mostly bacteria, but also fungi). It accumulates on the hard tissues of teeth in the oral cavity mainly due to improper oral hygiene and diet. Notably, the imbalance between the tooth biofilm and host response leads to the most common oral diseases such as dental caries and periodontal disease [159], and for this reason, the elimination of dental plaque results in beneficial local and systemic effects. Although a limited number of studies have been performed using dental plaque, research performed by both Leszczyńska et al. [143] and Niemirowicz et al. [137] confirm that even in the presence of accumulated dental plaque, therapeutic activity persists. Considering the example of *Candida* yeast, it might be stated that although the concentrations of ceragenins required to limit the outgrowth of pathogens in dental plaque are higher than those measured in PBS or even in the presence of saliva, MIC values are still within non-toxic and relatively low levels, i.e., ranging from 8 to 16 µg/mL. These results strongly suggest that an oral formulation containing ceragenin would be highly successful in eliminating harmful microorganisms from the mouth. Furthermore, this formulation would not be influenced by different body fluids, which would enhance its practicality in a clinical setting.

## 8. Recent Achievements in the Development of Ceragenin-Based Oral Formulations for the Prevention and Therapy of Oral Diseases

The use of ceragenins in the creation of formulations to effectively maintain oral hygiene, both as a preventive measure and for treatment purposes, is continuing to be explored. Over the last two years, two studies were published with potential applications. In the first, published by Tokajuk et al. in 2022 [158], the potential of ceragenin CSA-44 to control mature biofilms of *E. faecalis* and *C. albicans* and in vitro biofilm prevention on removed teeth and composite disks was examined. The study was motivated by two practical facts: (i) the necessity of repair or replacement of acrylic fillings after *C. albicans* infection and (ii) the correlation between biofilm formation on teeth and dental fillings, usually made of composite materials, with the development of caries and periodontal diseases. The authors demonstrated that upon administration of CSA-44, a significant decrease in clusters of biofilm structures was noted, which indicates the protective effects of CSA-44 against mono- and dual-species biofilms. A decrease in stiffness of CSA-44-treated biofilm was also recorded, suggesting it potentially facilitated the removal of the biofilm and possibly improved the penetration of other antimicrobials into the biofilm matrix. When examining the impact of microorganisms on dental composites, no discernible impact on the polymer structure or visible damage in the form of micro-cracks or other mechanical degradation caused by biofilm was observed. Our most recent study also emphasizes the potential use of ceragenins as components of oral mouthwashes to prevent/restrict the spread of oral infections [140]. This is achieved through the antimicrobial effect of ceragenins on planktonic and biofilm forms of *Candida* fungi, as well as anaerobic and aerobic oral disease-associated bacteria, such as *B. fragilis*, *E. faecalis*, and *S. mutans*. The research has shown that ceragenins CSA-13, CSA-44, CSA-131, and CSA-255 exhibit potent antimicrobial and antibiofilm effects against well-recognized representatives of bacterial and fungal pathogens found in the oral cavity. Notably, those effects were observed at a relatively low concentration of ceragenins of approx. 5–10 µg/mL, which are recognized as non-toxic against human gingival fibroblasts and human erythrocytes used as host-derived cells to assess ceragenin toxicity. Importantly, our study is the first to compare the effectiveness of ceragenin-containing solutions with six commercially available mouthwashes containing cetylpyridinium chloride, chlorhexidine digluconate, octenidine, or essential oils (with or without alcohol). Even though ceragenins exhibited comparable or slightly reduced efficacy relative to commercially available mouthwashes, they were distinguished by their lower toxicity toward host cells. This may potentially result in an improved maintenance of the stability of the host mucosal membrane [140]. These results, although carried out in in vitro settings, strongly suggest that ceragenins could be used in novel approaches for treatment of oral infections, and further research on the development of ceragenin-containing formulations is warranted.

## 9. Clinical Introduction of Ceragenins—Future Directions for Research on Ceragenin-Containing Oral Formulations

**Figure 3:**
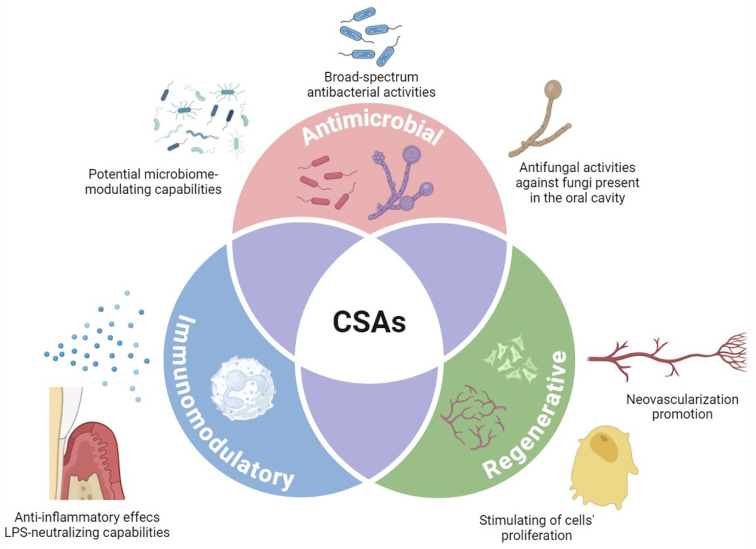
The multifaceted activities of ceragenins that make them a favorable candidate for further development of highly efficient formulations for oral hygiene and as therapeutics against oral diseases.

The extensive array of biological actions of ceragenins suggests several applications for ceragenins (Figure 3). Although a great majority of research regarding the antimicrobial and non-antimicrobial features of ceragenins was performed preclinically, some ceragenin-based solutions have entered a clinical research stage and are commercialized. Currently, the most advanced and clinically acceptable are ceragenin-coated endotracheal tubes designed to prevent the development of ventilator-associated pneumonia (VAP) in critically ill, high-risk patients and to significantly lower overall costs of hospital care [160]. Currently, CeraShield™ ETTs are approved for marketing in multiple countries, including Canada. Other applications include the use of ceragenins in dermal fillers, aimed to reduce erythema in esthetics injections, and as a chlorhexidine substitute in mouthwashes. In addition, other medical device-related techniques are being explored, including implants to prevent cardiac implantable electronic device (CIED) infections and coatings to reduce both implant-associated orthopedic infections and hemodialysis catheter-related infections. All these lines of research and favorable results from clinical trials strongly indicate the significant application potential of biomaterials, pharmaceutical formulations, and cosmetics containing ceragenins.

In the context of the further development of ceragenin-containing oral formulations, it is crucial to further expand the knowledge on potential beneficial features of ceragenins in experimental settings mimicking the environment of the oral cavity. Such an approach could help to answer the question of whether ceragenins might display beneficial effects in oral infection in mechanisms other than those that are directly antimicrobial, as demonstrated in multiple studies (Figure 3). Upon the demonstration of the anti-inflammatory properties of CSA-13, confirmed both in vitro (using LPS-stimulated human aorta endothelial cells) and in vivo (i.e., in mouse models of peritoneal infection [161] and *E.coli*-induced urinary tract infection [126]), a question is raised on whether ceragenins might diminish inflammation in infected oral cavity tissues. Ceragenin-mediated treatment would be beneficial for the treatment of both acute inflammation of the oral mucosa (including stomatitis or gingivitis) and chronic inflammation (during periodontitis). Previous studies have utilized human cathelicidin LL-37 to investigate its potential for treating periodontal tissue locally, namely in the subgingival area. According to two separate investigations, LL-37 inhibits LPS signaling while raising the production of two pro-inflammatory cytokines (IL-6 and IL-8) and promoting the synthesis of growth factors [162,163]. This suggests that the peptide (and possibly, other molecules mimicking its biological actions, like ceragenins) may have a significant function in the remodeling of periodontal tissue. This statement is also further strengthened by published reports on possible regenerative properties of ceragenins, demonstrating their ability to stimulate the migration of human keratinocytes and promote tube formation [136], as well as enhance human osteoblast proliferation [137]. Further research on the utility of ceragenins in the therapy of subjects who need periodontal regeneration and are undergoing surgical procedures and dental treatments is justified. It would also be beneficial to perform research on the impact of ceragenins on the composition of oral cavity microbiota, particularly in light of reports demonstrating the non-beneficial alterations of oral microbiome induced by chlorhexidine and commercial mouthwashes [164]. To date, the only studies on ceragenin-mediated changes in microbiota composition were performed by profiling microbe genera abundances in the gastrointestinal tract. As mentioned in previous sections, ceragenins have some bacterial selectivity, eradicating pathogenic bacteria and sparing beneficial organisms, although this topic needs to be more thoroughly explored.

Moreover, a thorough examination of local and systemic toxicity and the lifetime of ceragenins is critical for the clinical applicability of CSAs. It is well recognized that the toxicity and lifetime of AMPs resulting from their membrane-permeabilizing properties and the susceptibility of these peptides to proteases are important considerations in their development as therapeutic agents [165,166]. Particularly at higher concentrations, membrane-active compounds might result in hemolytic and cytotoxic activity [167,168]. Several strategies have been explored to address the toxicity issue of AMPs. One approach is using nanoparticles or other delivery systems to reduce the harmful effects of AMPs and other membrane-active molecules while maintaining their antimicrobial activity [169,170]. Favorable effects were also observed using poloxamer-based micelles, such as those formed from Pluronic F-127 or upon covalent immobilization to hydrogel-based soft contact lenses [171,172,173]. Available data strongly suggest that in such an aspect, ceragenins might offer several advantages over AMPs. Firstly, due to their non-peptide structure, they are less prone to degradation in body fluids, including serum [128]. Moreover, in a great majority of studies, including ours that was published most recently, ceragenins display antimicrobial effects at non-toxic doses of CSAs [140,174]. Nevertheless, it is crucial to expand the studies using oral cavity environment-mimicking experimental conditions.

## 10. Conclusions

Ceragenins, which are lipid analogs of naturally occurring AMPs, imitate their positive effects surpassing many of their advantageous biological features [169] and offering a promising and creative strategy for developing enhanced antimicrobial agents to combat both acute and chronic disorders in the oral cavity [140]. Particularly, when compared to endogenous AMPs, they display resistance to proteolytic degradation and are less susceptible to inhibitory effects of components of oral cavity fluids, including saliva or dental plaque [137]. In a great majority of studies, ceragenins effectively target a wide range of bacteria and fungi often found in the oral environment (Table 3), which makes them a compelling option for commercially available mouth disinfectants and antimicrobial compounds [128,144]. Their effectiveness was also confirmed against drug-resistant pathogens and their capability to induce pathogens’ resistance itself is recognized as low [175]. Moreover, the local administration of ceragenins would diminish any adverse effects of these compounds, even in the case of high-dose applications. Reports on the regenerative, immunomodulatory, and anti-inflammatory properties of ceragenins also suggest the potential usefulness of these compounds in dentistry (Figure 3). However, further investigation is needed as many of these biological characteristics have not been studied in laboratory and animal models that accurately replicate the complex composition and functions of the oral environment.

## Figures and Tables

**Table 1:** Results from the assessment of the safety and effectiveness of AMP-containing formulations against oral diseases in clinical trials (published results from the last 5 years).

Main Compound	Composition	Oral Disease/Medical Condition	Phase	Results	Ref.
**KSL-W**	Antiplaque chewing gum	Dental plaque regrowth	Phase 2	Inhibition of the regrowth of dental plaque over 4 days	[95]
**Probiotics**	Milk supplemented with *Lactobacillus rhamnosus* SP1	Caries in children	No data	Decrease in hBD-3 in saliva Decreased occurrence of caries in preschool children	[96]
**Nisin**	Guar gum (biogel)	Periodontal disease and dental plaque formation in dogs	Pilot study	Inhibit bacteria from canine dental plaque	[97]
**ε-PL, funme peptide, domiphen**	BOP^®^ mouthwash supplemented with AMPs	Halitosis Supragingival plaque	Phase 1	Lower levels of volatile sulfur compounds Decrease in plaque index	[75]
**AMPs**	Biokiller oral biological antimicrobial gel	Stage III grade B periodontitis	Phase 4	Reduced periodontal probing depth Decreased abundance of periodontal pathogens Increased abundance of periodontal probiotics	[93]
**P11-4**	Nanosilver fluoride varnish (NSF), P11-4 and sodium fluoride (NaF) varnish	Effect on salivary cariogenic bacteria	Not applicable	High activity of P11-4 after 1 month with sustained effect until 3 months	[98]

**Table 2:** Main similarities and differences between endogenous AMPs and ceragenins.

Characteristic	Antimicrobial Peptides	Ceragenins
Structure	Short peptides, typically < 100 amino acids	Non-peptide molecules based on the chemical structure of cholic acid
Charge	Cationic allowing to interact with negatively charged membranes	
Molecular weight (MW)	Mostly < 10 kDa	MW often < 1 kDa
Amphiphilic nature	Present	
Main mechanism of action	Targeting the microbial cell membrane leading to membrane disruption, depolarization, and cell death	
Spectrum of activity	A broad spectrum of activity against bacterial, fungal, and viral pathogens, including drug-resistant ones	
Potency of activity	High antimicrobial activities	In most cases, high activity, greater than those reported for AMPs or conventional antimicrobial
Susceptibility to proteases	Susceptible to proteolytic degradation	Resistant to proteases due to non-peptide chemical structure
Activity in the presence of body fluids	Varied, often inhibited by components of body fluids	Typically preserved
Hemolytic activity	Varied, sometimes detected in antimicrobial concentration ranges	Detected at higher concentrations, mostly non-hemolytic at antimicrobial concentration ranges
Ease of synthesis and purification	Costly ways of synthesis and purification	Easier and more cost-effective to synthesize

**Table 3:** Susceptibility of most clinically relevant oral cavity-habituating microbial pathogens to ceragenins.

Microorganism	CSA	Number of Tested Strains	MIC/MIC Range [µg/mL]	Refs.
*Bacteroides fragilis*	CSA-13/CSA-44/CSA-131/CSA-255	3 clinical strains	1–64	[140]
*Bacteroides thetaiotaomicron*	CSA-13/CSA-131	1 clinical strain	8	[124]
*Bacteroides stercoris*	CSA-13/CSA-131	1 clinical strain	2–8	[124]
*Candida albicans*	CSA-8/CSA-13/CSA-44/CSA-131/CSA-138/CSA-142/CSA-192/CSA-255	53 clinical strains, 3 laboratory strains	0.25–64	[128,140,141,142]
*Candida glabrata*	CSA-8/CSA-13/CSA-44/CSA-131/CSA-138/CSA-142/CSA-192/CSA-255	15 clinical strains	1–128	[140,141]
*Candida kefyr*	CSA-8/CSA-13/CSA-44/CSA-131/CSA-138/CSA-142/CSA-192	1 clinical strain	1–8	[141]
*Enterococcus faecalis*	CSA-13/CSA-44/CSA-90/CSA-92/CSA-131/CSA-255	4 laboratory strains	1.4–>128	[140,143]
Filamentous fungi	CSA-131/CSA-192	18 clinical and environmental isolates	1–16	[128]
*Streptococcus mutans*	CSA-13/CSA-90/CSA-92	24 clinical isolates, 1 laboratory strain	1–8	[143,144]
*Streptococcus salivarius*	CSA-13/CSA-90/CSA-92	1 laboratory strain	0.7–1.5	[143]
*Streptococcus sanguinis*	CSA-13/CSA-90/CSA-92	1 laboratory strain	0.7–1.6	[143]
*Streptococcus pneumoniae*	CSA-13/CSA-90/CSA-92	1 clinical strain	0.35–1.5	[143]
*Streptococcus pyogenes*	CSA-13/CSA-90/CSA-92	1 clinical strain	0.35–0.75	[143]
*Fusobacterium nucleatum*	CSA-13/CSA-90/CSA-92	1 laboratory strain	11.2–11.7	[143]
*Haemophilus influenzae*	CSA-13/CSA-90/CSA-92	1 clinical strain	0.35–0.7	[143]
*Lactobacillus casei* ssp. *casei*	CSA-13/CSA-90/CSA-92	1 laboratory strain	22.4–44.8	[143]
*Peptostreptococcus* spp.	CSA-13/CSA-131	1 clinical strain	0.5–4	[124]
*Porphyromonas gingivalis*	CSA-13/CSA-90/CSA-92	12 clinical isolates, 1 laboratory strain	2–16	[143,144]
*Porphyromonas cangingivalis*	CSA-13	2 clinical isolates	2–16	[144]
*Porphyromonas circumdentaria*	CSA-13	10 clinical isolates	1–2	[144]

## References

[1] Li X., Liu Y., Yang X., Li C., Song Z. (2022). The Oral Microbiota: Community Composition, Influencing Factors, Pathogenesis, and Interventions. Front. Microbiol..

[2] Wade W.G. (2013). The oral microbiome in health and disease. Pharmacol. Res..

[3] Palmer R.J. (2014). Composition and development of oral bacterial communities. Periodontol. 2000.

[4] Hayes R.B., Ahn J., Fan X., Peters B.A., Ma Y., Yang L., Agalliu I., Burk R.D., Ganly I., Purdue M.P. (2018). Association of Oral Microbiome with Risk for Incident Head and Neck Squamous Cell Cancer. JAMA Oncol..

[5] Rodríguez-Lozano B., González-Febles J., Garnier-Rodríguez J.L., Dadlani S., Bustabad-Reyes S., Sanz M., Sánchez-Alonso F., Sánchez-Piedra C., González-Dávila E., Díaz-González F. (2019). Association between severity of periodontitis and clinical activity in rheumatoid arthritis patients: A case-control study. Arthritis Res. Ther..

[6] Czesnikiewicz-Guzik M., Osmenda G., Siedlinski M., Nosalski R., Pelka P., Nowakowski D., Wilk G., Mikolajczyk T.P., Schramm-Luc A., Furtak A. (2019). Causal association between periodontitis and hypertension: Evidence from Mendelian randomization and a randomized controlled trial of non-surgical periodontal therapy. Eur. Heart J..

[7] Peng X., Cheng L., You Y., Tang C., Ren B., Li Y., Xu X., Zhou X. (2022). Oral microbiota in human systematic diseases. Int. J. Oral Sci..

[8] Conrads G., About I. (2018). Pathophysiology of Dental Caries.

[9] Tokajuk J., Deptuła P., Piktel E., Daniluk T., Chmielewska S., Wollny T., Wolak P., Fiedoruk K., Bucki R. (2022). Cathelicidin LL-37 in Health and Diseases of the Oral Cavity. Biomedicines.

[10] Bechinger B., Gorr S.U. (2017). Antimicrobial Peptides: Mechanisms of Action and Resistance. J. Dent. Res..

[11] Khurshid Z., Naseem M., Sheikh Z., Najeeb S., Shahab S., Zafar M.S. (2016). Oral antimicrobial peptides: Types and role in the oral cavity. Saudi Pharm. J..

[12] Di Stefano M., Polizzi A., Santonocito S., Romano A., Lombardi T., Isola G. (2022). Impact of Oral Microbiome in Periodontal Health and Periodontitis: A Critical Review on Prevention and Treatment. Int. J. Mol. Sci..

[13] Sharma N., Bhatia S., Sodhi A.S., Batra N. (2018). Oral microbiome and health. AIMS Microbiol..

[14] Segata N., Haake S.K., Mannon P., Lemon K.P., Waldron L., Gevers D., Huttenhower C., Izard J. (2012). Composition of the adult digestive tract bacterial microbiome based on seven mouth surfaces, tonsils, throat and stool samples. Genome Biol..

[15] Chen Y., Huang Z., Tang Z., Huang Y., Huang M., Liu H., Ziebolz D., Schmalz G., Jia B., Zhao J. (2022). More Than Just a Periodontal Pathogen -the Research Progress on *Fusobacterium nucleatum*. Front. Cell. Infect. Microbiol..

[16] Caufield P.W., Schön C.N., Saraithong P., Li Y., Argimón S. (2015). Oral Lactobacilli and Dental Caries: A Model for Niche Adaptation in Humans. J. Dent. Res..

[17] Xu H., Hao W., Zhou Q., Wang W., Xia Z., Liu C., Chen X., Qin M., Chen F. (2014). Plaque bacterial microbiome diversity in children younger than 30 months with or without caries prior to eruption of second primary molars. PLoS ONE.

[18] Kahharova D., Pappalardo V.Y., Buijs M.J., de Menezes R.X., Peters M., Jackson R., Hara A.T., Eckert G., Katz B., Keels M.A. (2023). Microbial Indicators of Dental Health, Dysbiosis, and Early Childhood Caries. J. Dent. Res..

[19] Zheng H., Xie T., Li S., Qiao X., Lu Y., Feng Y. (2021). Analysis of oral microbial dysbiosis associated with early childhood caries. BMC Oral Health.

[20] Aas J.A., Griffen A.L., Dardis S.R., Lee A.M., Olsen I., Dewhirst F.E., Leys E.J., Paster B.J. (2008). Bacteria of dental caries in primary and permanent teeth in children and young adults. J. Clin. Microbiol..

[21] Becker M.R., Paster B.J., Leys E.J., Moeschberger M.L., Kenyon S.G., Galvin J.L., Boches S.K., Dewhirst F.E., Griffen A.L. (2002). Molecular analysis of bacterial species associated with childhood caries. J. Clin. Microbiol..

[22] Xu H., Tian J., Hao W., Zhang Q., Zhou Q., Shi W., Qin M., He X., Chen F. (2018). Oral Microbiome Shifts From Caries-Free to Caries-Affected Status in 3-Year-Old Chinese Children: A Longitudinal Study. Front. Microbiol..

[23] Sanz M., Marco Del Castillo A., Jepsen S., Gonzalez-Juanatey J.R., D’Aiuto F., Bouchard P., Chapple I., Dietrich T., Gotsman I., Graziani F. (2020). Periodontitis and cardiovascular diseases: Consensus report. J. Clin. Periodontol..

[24] Borgnakke W.S., Ylöstalo P.V., Taylor G.W., Genco R.J. (2013). Effect of periodontal disease on diabetes: Systematic review of epidemiologic observational evidence. J. Periodontol..

[25] Nwizu N., Wactawski-Wende J., Genco R.J. (2020). Periodontal disease and cancer: Epidemiologic studies and possible mechanisms. Periodontol. 2000.

[26] Vieira Colombo A.P., Magalhães C.B., Hartenbach F.A., Martins do Souto R., Maciel da Silva-Boghossian C. (2016). Periodontal-disease-associated biofilm: A reservoir for pathogens of medical importance. Microb. Pathog..

[27] Liu S., Xie G., Chen M., He Y., Yu W., Chen X., Mao W., Liu N., Zhang Y., Chang Q. (2023). Oral microbial dysbiosis in patients with periodontitis and chronic obstructive pulmonary disease. Front. Cell. Infect. Microbiol..

[28] Loesche W.J., Grossman N.S. (2001). Periodontal disease as a specific, albeit chronic, infection: Diagnosis and treatment. Clin. Microbiol. Rev..

[29] Zheng H., Xu L., Wang Z., Li L., Zhang J., Zhang Q., Chen T., Lin J., Chen F. (2015). Subgingival microbiome in patients with healthy and ailing dental implants. Sci. Rep..

[30] Gao L., Xu T., Huang G., Jiang S., Gu Y., Chen F. (2018). Oral microbiomes: More and more importance in oral cavity and whole body. Protein Cell.

[31] Robinson C.M., Pfeiffer J.K. (2014). Viruses and the Microbiota. Annu. Rev. Virol..

[32] Ichinohe T., Pang I.K., Kumamoto Y., Peaper D.R., Ho J.H., Murray T.S., Iwasaki A. (2011). Microbiota regulates immune defense against respiratory tract influenza A virus infection. Proc. Natl. Acad. Sci. USA.

[33] Guarino A., Canani R.B., Spagnuolo M.I., Albano F., Di Benedetto L. (1997). Oral bacterial therapy reduces the duration of symptoms and of viral excretion in children with mild diarrhea. J. Pediatr. Gastroenterol. Nutr..

[34] Meyer J.E., Harder J., Sipos B., Maune S., Klöppel G., Bartels J., Schröder J.M., Gläser R. (2008). Psoriasin (S100A7) is a principal antimicrobial peptide of the human tongue. Mucosal Immunol..

[35] Kamekura R., Imai R., Takano K., Yamashita K., Jitsukawa S., Nagaya T., Ito F., Hirao M., Tsubota H., Himi T. (2016). Expression and Localization of Human Defensins in Palatine Tonsils. Adv. Otorhinolaryngol..

[36] Sahasrabudhe K.S., Kimball J.R., Morton T.H., Weinberg A., Dale B.A. (2000). Expression of the antimicrobial peptide, human beta-defensin 1, in duct cells of minor salivary glands and detection in saliva. J. Dent. Res..

[37] Dale B.A., Kimball J.R., Krisanaprakornkit S., Roberts F., Robinovitch M., O’Neal R., Valore E.V., Ganz T., Anderson G.M., Weinberg A. (2001). Localized antimicrobial peptide expression in human gingiva. J. Periodontal. Res..

[38] Bucki R., Leszczyńska K., Namiot A., Sokołowski W. (2010). Cathelicidin LL-37: A multitask antimicrobial peptide. Arch. Immunol. Ther. Exp..

[39] Lundy F.T., Irwin C.R., McLean D.F., Linden G.J., El Karim I.A. (2020). Natural Antimicrobials in the Dental Pulp. J. Endod..

[40] Mathews M., Jia H.P., Guthmiller J.M., Losh G., Graham S., Johnson G.K., Tack B.F., McCray P.B. (1999). Production of beta-defensin antimicrobial peptides by the oral mucosa and salivary glands. Infect. Immun..

[41] Waniczek D., Świętochowska E., Śnietura M., Kiczmer P., Lorenc Z., Muc-Wierzgoń M. (2022). Salivary Concentrations of Chemerin, α-Defensin 1, and TNF-α as Potential Biomarkers in the Early Diagnosis of Colorectal Cancer. Metabolites.

[42] Pei F., Wang M., Wang Y., Pan X., Cen X., Huang X., Jin Y., Zhao Z. (2022). Quantitative proteomic analysis of gingival crevicular fluids to identify novel biomarkers of gingival recession in orthodontic patients. J. Proteom..

[43] Khurshid Z., Zafar M.S., Naseem M., Khan R.S., Najeeb S. (2018). Human Oral Defensins Antimicrobial Peptides: A Future Promising Antimicrobial Drug. Curr. Pharm. Des..

[44] Wang Z., Shen Y., Haapasalo M. (2017). Antibiofilm peptides against oral biofilms. J. Oral Microbiol..

[45] Cai J., Li X., Du H., Jiang C., Xu S., Cao Y. (2020). Immunomodulatory significance of natural peptides in mammalians: Promising agents for medical application. Immunobiology.

[46] Mai S., Mauger M.T., Niu L.N., Barnes J.B., Kao S., Bergeron B.E., Ling J.Q., Tay F.R. (2017). Potential applications of antimicrobial peptides and their mimics in combating caries and pulpal infections. Acta Biomater..

[47] Lynge Pedersen A.M., Belstrøm D. (2019). The role of natural salivary defences in maintaining a healthy oral microbiota. J. Dent..

[48] Winter J., Jepsen S. (2024). Role of innate host defense proteins in oral cancerogenesis. Periodontol. 2000.

[49] Dale B.A., Fredericks L.P. (2005). Antimicrobial peptides in the oral environment: Expression and function in health and disease. Curr. Issues Mol. Biol..

[50] Hans M., Madaan Hans V. (2014). Epithelial antimicrobial peptides: Guardian of the oral cavity. Int. J. Pept..

[51] Niu J.Y., Yin I.X., Mei M.L., Wu W.K.K., Li Q.L., Chu C.H. (2021). The multifaceted roles of antimicrobial peptides in oral diseases. Mol. Oral Microbiol..

[52] Morio K.A., Sternowski R.H., Brogden K.A. (2023). Induction of Endogenous Antimicrobial Peptides to Prevent or Treat Oral Infection and Inflammation. Antibiotics.

[53] Ghosh S.K., Man Y., Fraiwan A., Waters C., McKenzie C., Lu C., Pfau D., Kawsar H., Bhaskaran N., Pandiyan P. (2024). Beta-defensin index: A functional biomarker for oral cancer detection. Cell Rep. Med..

[54] Kawsar H.I., Weinberg A., Hirsch S.A., Venizelos A., Howell S., Jiang B., Jin G. (2009). Overexpression of human beta-defensin-3 in oral dysplasia: Potential role in macrophage trafficking. Oral Oncol..

[55] Zhang C., Han Y., Miao L., Yue Z., Xu M., Liu K., Hou J. (2023). Human β-defensins are correlated with the immune infiltration and regulated by vitamin D. J. Periodontal Res..

[56] Bissell J., Joly S., Johnson G.K., Organ C.C., Dawson D., McCray P.B., Guthmiller J.M. (2004). Expression of beta-defensins in gingival health and in periodontal disease. J. Oral Pathol. Med..

[57] Soldati K.R., Gutierrez L.S., Anovazzi G., Scarel-Caminaga R.M., Zandim-Barcelos D.L. (2022). Impact of smoking on protein levels of beta-defensins in periodontal disease. Braz. Dent. J..

[58] Nireeksha, Hegde M.N., Kumari N S. (2024). Potential role of salivary vitamin D antimicrobial peptide LL-37 and interleukins in severity of dental caries: An exvivo study. BMC Oral Health.

[59] Tao R., Jurevic R.J., Coulton K.K., Tsutsui M.T., Roberts M.C., Kimball J.R., Wells N., Berndt J., Dale B.A. (2005). Salivary antimicrobial peptide expression and dental caries experience in children. Antimicrob. Agents Chemother..

[60] Rm V.R., Singh N., Murmu S., Abhishek, Raina S., Singh S. (2023). Salivary physicochemical characteristics and antimicrobial human peptide among Indian children with dental caries. Bioinformation.

[61] Nittayananta W., Lerdsamran H., Chutiwitoonchai N., Promsong A., Srichana T., Netsomboon K., Prasertsopon J., Kerdto J. (2024). A novel film spray containing curcumin inhibits SARS-CoV-2 and influenza virus infection and enhances mucosal immunity. Virol. J..

[62] Campbell J.X., Schulte N.B., Lai B., Harris H.H., Franz K.J. (2023). Histatin-5 interacts with cellular copper to promote antifungal activity against Candida albicans. Metallomics.

[63] Stewart L.J., Hong Y., Holmes I.R., Firth S.J., Ahmed Y., Quinn J., Santos Y., Cobb S.L., Jakubovics N.S., Djoko K.Y. (2023). Salivary Antimicrobial Peptide Histatin-5 Does Not Display Zn(II)-Dependent or -Independent Activity against Streptococci. ACS Infect. Dis..

[64] Turkoglu O., Emingul G., Eren G., Atmaca H., Kutukculer N., Atilla G. (2017). Levels of ll-37 antimicrobial peptide in the gingival crevicular fluid of young and middle-aged subjects with or without gingivitis. J. Istanb. Univ. Fac. Dent..

[65] Lappin M.J., Dellett M., Mills K.I., Lundy F.T., Irwin C.R. (2023). The neutralising and stimulatory effects of antimicrobial peptide LL-37 in human gingival fibroblasts. Arch. Oral Biol..

[66] Contini C., Fadda L., Lai G., Masala C., Olianas A., Castagnola M., Messana I., Iavarone F., Bizzarro A., Masullo C. (2024). A top-down proteomic approach reveals a salivary protein profile able to classify Parkinson’s disease with respect to Alzheimer’s disease patients and to healthy controls. Proteomics.

[67] Seminario A.L., Karczewski A.E., Chung W., Wang Y., Wamalwa D., Benki-Nugent S., John-Stewart G., Slyker J.A., Kemoli A. (2024). Salivary Cathelicidin (LL-37) in Children and Adolescents Living with HIV. Biomed. Hub.

[68] Mohtasham N., Bargi R., Farshbaf A., Shahri M.V., Hesari K.K., Mohajertehran F. (2023). Salivary Antiviral and Antibacterial Properties in the Encounter of SARS-CoV-2. Curr. Pharm. Des..

[69] Abdul-Kareem H.H., Al-Maqtoofi M.Y., Burghal A.A. (2023). Impact of COVID-19 vaccination on saliva immune barriers: IgA, lysozyme, and lactoferrin. Arch. Virol..

[70] Guglielmi F., Kirschner F., Staderini E., Iavarone F., Fiorino A., Gallenzi P. (2023). Proteomic analysis of salivary inflammatory biomarkers of developmental gingival enlargements in patients with West and Noonan syndromes: A preliminary pilot single-center retrospective study. Eur. Rev. Med. Pharmacol. Sci..

[71] Zhan L. (2018). Rebalancing the Caries Microbiome Dysbiosis: Targeted Treatment and Sugar Alcohols. Adv. Dent. Res..

[72] Zeng Y., Fadaak A., Alomeir N., Wu Y., Wu T.T., Qing S., Xiao J. (2023). Effect of Probiotic. Int. J. Mol. Sci..

[73] Choudhary P., Kraatz H.B., Lévesque C.M., Gong S.G. (2023). Microencapsulation of Probiotic. ACS Omega.

[74] Nogueira M.B., Massaut K.B., Vitola H.R.S., Siqueira M.F.F., da Silva W.P., Fiorentini Â. (2023). Antagonistic activity of *Lactobacillus* spp. and *Bifidobacterium* spp. against cariogenic Streptococcus mutans in vitro and viability when added to chewing gum during storage. Braz. J. Microbiol..

[75] Shen S., Liu X., Huang J., Sun Y., Liu B., Song W., Meng L., Du M., Feng Q. (2024). Efficacy of a mouthwash containing ε-poly-L-lysine, funme peptides and domiphen in reducing halitosis and supragingival plaque: A randomized clinical trial. BMC Oral Health.

[76] Zhang Y., Chen Y., Liu Z., Peng X., Lu J., Wang K., Zhang L. (2023). Encapsulation of a novel peptide derived from histatin-1 in liposomes against initial enamel caries in vitro and in vivo. Clin. Oral Investig..

[77] Golshani S., Vatanara A., Balalaie S., Kadkhoda Z., Abdollahi M., Amin M. (2023). Development of a Novel Histatin-5 Mucoadhesive Gel for the Treatment of Oral Mucositis: In Vitro Characterization and In Vivo Evaluation. AAPS PharmSciTech.

[78] Zhou S., Miao D., Wen J., Zhang Q., Hu D., Liu N., Li J., Zhang Y., Wang K., Chen Y. (2024). Microcin C7-laden modified gelatin based biocomposite hydrogel for the treatment of periodontitis. Int. J. Biol. Macromol..

[79] Chen M., Hu Z., Shi J., Xie Z. (2024). Human β-defensins and their synthetic analogs: Natural defenders and prospective new drugs of oral health. Life Sci..

[80] Kompuinen J., Keskin M., Yilmaz D., Gürsoy M., Gürsoy U.K. (2023). Human β-Defensins in Diagnosis of Head and Neck Cancers. Cells.

[81] Qi S., Zhao S., Zhang H., Liu S., Liu J., Yang J., Qi Y., Zhao Q., Jin Y., Wang F. (2023). Novel casein antimicrobial peptides for the inhibition of oral pathogenic bacteria. Food Chem..

[82] Sarkar T., Chetia M., Chatterjee S. (2021). Antimicrobial Peptides and Proteins: From Nature’s Reservoir to the Laboratory and Beyond. Front. Chem..

[83] Vanzolini T., Bruschi M., Rinaldi A.C., Magnani M., Fraternale A. (2022). Multitalented Synthetic Antimicrobial Peptides and Their Antibacterial, Antifungal and Antiviral Mechanisms. Int. J. Mol. Sci..

[84] Vasconcelos M.A., da Silva B.R., Andrade A.L., de Azevedo Pinheiro A., Evaristo F.F.V., Arruda F.V.S., Lorenzón E.N., Cilli E.M., Teixeira E.H. (2023). Antimicrobial and Antibiofilm Activity of Synthetic Peptide [W7]KR12-KAEK Against Enterococcus faecalis Strains. Curr. Microbiol..

[85] de la Fuente-Núñez C., Reffuveille F., Mansour S.C., Reckseidler-Zenteno S.L., Hernández D., Brackman G., Coenye T., Hancock R.E. (2015). D-enantiomeric peptides that eradicate wild-type and multidrug-resistant biofilms and protect against lethal Pseudomonas aeruginosa infections. Chem. Biol..

[86] Wang D., Shen Y., Ma J., Hancock R.E.W., Haapasalo M. (2017). Antibiofilm Effect of D-enantiomeric Peptide Alone and Combined with EDTA In Vitro. J. Endod..

[87] Hu J., Yu J., Liu H., Wang Z., Haapasalo M., Haney E.F., Hancock R.E.W., Deng S., Shen Y. (2023). Dynamic killing effectiveness of mouthrinses and a d-enantiomeric peptide on oral multispecies biofilms grown on dental restorative material surfaces. J. Dent..

[88] Luo J., Feng Z., Jiang W., Jiang X., Chen Y., Lv X., Zhang L. (2021). Novel lactotransferrin-derived synthetic peptides suppress cariogenic bacteria. J. Oral Microbiol..

[89] Feng Z., Luo J., Lyu X., Chen Y., Zhang L. (2022). Selective antibacterial activity of a novel lactotransferrin-derived antimicrobial peptide LF-1 against Streptococcus mutans. Arch. Oral Biol..

[90] Luo J., Feng Z., Lyu X., Zhang L. (2023). Novel Lactotransferrin-Derived Antimicrobial Peptide LF-1 Inhibits the Cariogenic Virulence Factors of. Antibiotics.

[91] Zhang O.L., Niu J.Y., Yin I.X., Yu O.Y., Mei M.L., Chu C.H. (2023). Antibacterial Properties of the Antimicrobial Peptide Gallic Acid-Polyphemusin I (GAPI). Antibiotics.

[92] Soldati K.R., Jiang Y., Brandt B.W., Exterkate R.A.M., Buijs M.J., Nazmi K., Kaman W.E., Cheng L., Bikker F.J., Crielaard W. (2023). Differential Modulation of Saliva-Derived Microcosm Biofilms by Antimicrobial Peptide LL-31 and D-LL-31. Pathogens.

[93] Xiang S., Han N., Xie Y., Du J., Luo Z., Xu J., Liu Y. (2024). Antimicrobial peptides in treatment of Stage III Grade B periodontitis: A randomized clinical trial. Oral Dis..

[94] Barbour A., Smith L., Oveisi M., Williams M., Huang R.C., Marks C., Fine N., Sun C., Younesi F., Zargaran S. (2023). Discovery of phosphorylated lantibiotics with proimmune activity that regulate the oral microbiome. Proc. Natl. Acad. Sci. USA.

[95] Kirkwood B., Miller M., Milleman J., Milleman K., Leung K. (2020). Four-day plaque regrowth evaluation of a peptide chewing gum in a double-blind randomized clinical trial. Clin. Exp. Dent. Res..

[96] Sandoval F., Faleiros S., Cabello R., Díaz-Dosque M., Rodríguez G., Escobar A. (2021). The consumption of milk supplemented with probiotics decreases the occurrence of caries and the salivary concentration of hβD-3 in children. Clin. Oral Investig..

[97] Cunha E., Valente S., Nascimento M., Pereira M., Tavares L., Dias R., Oliveira M. (2021). Influence of the dental topical application of a nisin-biogel in the oral microbiome of dogs: A pilot study. PeerJ.

[98] Atteya S.M., Amer H.A., Saleh S.M., Safwat Y. (2024). The effect of nano silver fluoride, self-assembling peptide and sodium fluoride varnish on salivary cariogenic bacteria: A randomized controlled clinical trial. Clin. Oral Investig..

[99] Jennings M.C., Minbiole K.P., Wuest W.M. (2015). Quaternary Ammonium Compounds: An Antimicrobial Mainstay and Platform for Innovation to Address Bacterial Resistance. ACS Infect. Dis..

[100] Kang H.K., Kim C., Seo C.H., Park Y. (2017). The therapeutic applications of antimicrobial peptides (AMPs): A patent review. J. Microbiol..

[101] Amer L., Retout M., Jokerst J.V. (2024). Activatable prodrug for controlled release of an antimicrobial peptide via the proteases overexpressed in. Theranostics.

[102] Dai J., Fischer N.G., Rahimi J.R., Wang H., Hu C., Chen W., Lin Y., Sang T., Chew H.P., Kong L. (2024). Interpenetrating nanofibrillar membrane of self-assembled collagen and antimicrobial peptides for enhanced bone regeneration. Int. J. Biol. Macromol..

[103] Agarwal V., Tikhonov A., Metlitskaya A., Severinov K., Nair S.K. (2012). Structure and function of a serine carboxypeptidase adapted for degradation of the protein synthesis antibiotic microcin C7. Proc. Natl. Acad. Sci. USA.

[104] Toledano-Osorio M., Osorio R., Bueno J., Vallecillo C., Vallecillo-Rivas M., Sanz M. (2024). Next-generation antibacterial nanopolymers for treating oral chronic inflammatory diseases of bacterial origin. Int. Endod. J..

[105] Garcia de Carvalho G., Maquera-Huacho P.M., Silva Pontes C., Annunzio S.R., Fontana Mendonça C.R., Nara de Souza Rastelli A., de Oliveira K.T., Teughels W., Chorilli M., Leal Zandim-Barcelos D. (2023). Chlorin-e6 conjugated to the antimicrobial peptide LL-37 loaded nanoemulsion enhances photodynamic therapy against multi-species biofilms related to periodontitis. Photodiagnosis Photodyn. Ther..

[106] Li K., Tang Z., Song K., Fischer N.G., Wang H., Guan Y., Deng Y., Cai H., Hassan S.U., Ye Z. (2023). Multifunctional nanocoating for enhanced titanium implant osseointegration. Colloids Surf. B Biointerfaces.

[107] Lin H.J., Huang T.C., Muthusamy S., Lee J.F., Duann Y.F., Lin C.H. (2012). Piscidin-1, an antimicrobial peptide from fish (hybrid striped bass morone saxatilis x M. chrysops), induces apoptotic and necrotic activity in HT1080 cells. Zoolog. Sci..

[108] Chiu F.C., Kuo H.M., Yu C.L., Selvam P., Su I.L., Tseng C.C., Yuan C.H., Wen Z.H. (2024). Marine-derived antimicrobial peptide piscidin-1 triggers extrinsic and intrinsic apoptosis in oral squamous cell carcinoma through reactive oxygen species production and inhibits angiogenesis. Free. Radic. Biol. Med..

[109] Xu H., Zhang F., Wang M., Lv H., Yu D.G., Liu X., Shen H. (2022). Electrospun hierarchical structural films for effective wound healing. Biomater Adv..

[110] Yap K.M., Sekar M., Fuloria S., Wu Y.S., Gan S.H., Mat Rani N.N.I., Subramaniyan V., Kokare C., Lum P.T., Begum M.Y. (2021). Drug Delivery of Natural Products Through Nanocarriers for Effective Breast Cancer Therapy: A Comprehensive Review of Literature. Int. J. Nanomed..

[111] Yuan Z., Sheng D., Jiang L., Shafiq M., Khan A.U.R., Hashim R., Chen Y., Li B., Xie X., Chen J. (2022). Vascular Endothelial Growth Factor-Capturing Aligned Electrospun Polycaprolactone/Gelatin Nanofibers Promote Patellar Ligament Regeneration. Acta Biomater..

[112] Brimo N., Serdaroğlu D., Uysal B. (2022). Comparing Antibiotic Pastes with Electrospun Nanofibers as Modern Drug Delivery Systems for Regenerative Endodontics. Curr. Drug Deliv..

[113] Zhao P., Chen W., Feng Z., Liu Y., Liu P., Xie Y., Yu D.G. (2022). Electrospun Nanofibers for Periodontal Treatment: A Recent Progress. Int. J. Nanomed..

[114] Palasuk J., Kamocki K., Hippenmeyer L., Platt J.A., Spolnik K.J., Gregory R.L., Bottino M.C. (2014). Bimix antimicrobial scaffolds for regenerative endodontics. J. Endod..

[115] Yang F., Miao Y., Wang Y., Zhang L.M., Lin X. (2017). Electrospun Zein/Gelatin Scaffold-Enhanced Cell Attachment and Growth of Human Periodontal Ligament Stem Cells. Materials.

[116] Wang Y., Liu Y., Zhang X., Liu N., Yu X., Gao M., Wang W., Wu T. (2021). Engineering Electrospun Nanofibers for the Treatment of Oral Diseases. Front. Chem..

[117] Jin S., Yang R., Hu C., Xiao S., Zuo Y., Man Y., Li Y., Li J. (2023). Plant-Derived Polyphenol and LL-37 Peptide-Modified Nanofibrous Scaffolds for Promotion of Antibacterial Activity, Anti-Inflammation, and Type-H Vascularized Bone Regeneration. ACS Appl. Mater. Interfaces.

[118] Edmans J.G., Murdoch C., Santocildes-Romero M.E., Hatton P.V., Colley H.E., Spain S.G. (2020). Incorporation of lysozyme into a mucoadhesive electrospun patch for rapid protein delivery to the oral mucosa. Mater. Sci. Eng. C Mater. Biol. Appl..

[119] He Y., Jin Y., Wang X., Yao S., Li Y., Wu Q., Ma G., Cui F., Liu H. (2018). An Antimicrobial Peptide-Loaded Gelatin/Chitosan Nanofibrous Membrane Fabricated by Sequential Layer-by-Layer Electrospinning and Electrospraying Techniques. Nanomaterials.

[120] Savage P.B., Li C., Taotafa U., Ding B., Guan Q. (2002). Antibacterial properties of cationic steroid antibiotics. FEMS Microbiol. Lett..

[121] Hashemi M.M., Rovig J., Holden B.S., Taylor M.F., Weber S., Wilson J., Hilton B., Zaugg A.L., Ellis S.W., Yost C.D. (2018). Ceragenins are active against drug-resistant Candida auris clinical isolates in planktonic and biofilm forms. J. Antimicrob. Chemother.

[122] Assoni L., Milani B., Carvalho M.R., Nepomuceno L.N., Waz N.T., Guerra M.E.S., Converso T.R., Darrieux M. (2020). Resistance Mechanisms to Antimicrobial Peptides in Gram-Positive Bacteria. Front. Microbiol..

[123] Schindeler A., Yu N.Y., Cheng T.L., Sullivan K., Mikulec K., Peacock L., Matthews R., Little D.G. (2015). Local delivery of the cationic steroid antibiotic CSA-90 enables osseous union in a rat open fracture model of Staphylococcus aureus infection. J. Bone Jt. Surg. Am..

[124] Durnaś B., Piktel E., Wątek M., Wollny T., Góźdź S., Smok-Kalwat J., Niemirowicz K., Savage P.B., Bucki R. (2017). Anaerobic bacteria growth in the presence of cathelicidin LL-37 and selected ceragenins delivered as magnetic nanoparticles cargo. BMC Microbiol..

[125] Chin J.N., Jones R.N., Sader H.S., Savage P.B., Rybak M.J. (2008). Potential synergy activity of the novel ceragenin, CSA-13, against clinical isolates of Pseudomonas aeruginosa, including multidrug-resistant P. aeruginosa. J. Antimicrob. Chemother..

[126] Wnorowska U., Piktel E., Deptuła P., Wollny T., Król G., Głuszek K., Durnaś B., Pogoda K., Savage P.B., Bucki R. (2022). Ceragenin CSA-13 displays high antibacterial efficiency in a mouse model of urinary tract infection. Sci. Rep..

[127] Karasiński M., Wnorowska U., Durnaś B., Król G., Daniluk T., Skłodowski K., Głuszek K., Piktel E., Okła S., Bucki R. (2023). Ceragenins and Ceragenin-Based Core-Shell Nanosystems as New Antibacterial Agents against Gram-Negative Rods Causing Nosocomial Infections. Pathogens.

[128] Durnas B., Wnorowska U., Pogoda K., Deptula P., Watek M., Piktel E., Gluszek S., Gu X., Savage P.B., Niemirowicz K. (2016). Candidacidal Activity of Selected Ceragenins and Human Cathelicidin LL-37 in Experimental Settings Mimicking Infection Sites. PLoS ONE.

[129] Howell M.D., Streib J.E., Kim B.E., Lesley L.J., Dunlap A.P., Geng D., Feng Y., Savage P.B., Leung D.Y. (2009). Ceragenins: A class of antiviral compounds to treat orthopox infections. J. Investig. Dermatol..

[130] Ding B., Yin N., Liu Y., Cardenas-Garcia J., Evanson R., Orsak T., Fan M., Turin G., Savage P.B. (2004). Origins of cell selectivity of cationic steroid antibiotics. J. Am. Chem. Soc..

[131] Epand R.F., Savage P.B., Epand R.M. (2007). Bacterial lipid composition and the antimicrobial efficacy of cationic steroid compounds (Ceragenins). Biochim. Biophys. Acta.

[132] Epand R.F., Pollard J.E., Wright J.O., Savage P.B., Epand R.M. (2010). Depolarization, bacterial membrane composition, and the antimicrobial action of ceragenins. Antimicrob. Agents Chemother..

[133] Chmielewska S.J., Skłodowski K., Piktel E., Suprewicz Ł., Fiedoruk K., Daniluk T., Wolak P., Savage P.B., Bucki R. (2020). NDM-1 Carbapenemase-Producing Enterobacteriaceae are Highly Susceptible to Ceragenins CSA-13, CSA-44, and CSA-131. Infect. Drug Resist..

[134] Prasad S.V., Fiedoruk K., Zakrzewska M., Savage P.B., Bucki R. (2023). Glyoxylate Shunt and Pyruvate-to-Acetoin Shift Are Specific Stress Responses Induced by Colistin and Ceragenin CSA-13 in Enterobacter hormaechei ST89. Microbiol. Spectr..

[135] Wnorowska U., Łysik D., Piktel E., Zakrzewska M., Okła S., Lesiak A., Spałek J., Mystkowska J., Savage P.B., Janmey P. (2024). Ceragenin-mediated disruption of Pseudomonas aeruginosa biofilms. PLoS ONE.

[136] Olekson M.A., You T., Savage P.B., Leung K.P. (2017). Antimicrobial ceragenins inhibit biofilms and affect mammalian cell viability and migration in vitro. FEBS Open Bio.

[137] Niemirowicz K., Durnaś B., Tokajuk G., Piktel E., Michalak G., Gu X., Kułakowska A., Savage P.B., Bucki R. (2017). Formulation and candidacidal activity of magnetic nanoparticles coated with cathelicidin LL-37 and ceragenin CSA-13. Sci. Rep..

[138] Bucki R., Sostarecz A.G., Byfield F.J., Savage P.B., Janmey P.A. (2007). Resistance of the antibacterial agent ceragenin CSA-13 to inactivation by DNA or F-actin and its activity in cystic fibrosis sputum. J. Antimicrob. Chemother..

[139] Suprewicz Ł., Szczepański A., Lenart M., Piktel E., Fiedoruk K., Barreto-Duran E., Kula-Pacurar A., Savage P.B., Milewska A., Bucki R. (2023). Ceragenins exhibit antiviral activity against SARS-CoV-2 by increasing the expression and release of type I interferons upon activation of the host’s immune response. Antivir. Res..

[140] Czarnowski M., Słowińska M., Sawieljew M., Wnorowska U., Daniluk T., Król G., Karasiński M., Okła S., Savage P.B., Piktel E. (2024). Efficacy of Ceragenins in Controlling the Growth of Oral Microorganisms: Implications for Oral Hygiene Management. Pharmaceuticals.

[141] Hacioglu M., Guzel C.B., Savage P.B., Tan A.S.B. (2019). Antifungal susceptibilities, in vitro production of virulence factors and activities of ceragenins against *Candida* spp. isolated from vulvovaginal candidiasis. Med. Mycol..

[142] Bozkurt-Guzel C., Hacioglu M., Savage P.B. (2018). Investigation of the in vitro antifungal and antibiofilm activities of ceragenins CSA-8, CSA-13, CSA-44, CSA-131, and CSA-138 against Candida species. Diagn. Microbiol. Infect. Dis..

[143] Leszczynska K., Namiot D., Byfield F.J., Cruz K., Zendzian-Piotrowska M., Fein D.E., Savage P.B., Diamond S., McCulloch C.A., Janmey P.A. (2013). Antibacterial activity of the human host defence peptide LL-37 and selected synthetic cationic lipids against bacteria associated with oral and upper respiratory tract infections. J. Antimicrob. Chemother..

[144] Isogai E., Isogai H., Takahashi K., Okumura K., Savage P.B. (2009). Ceragenin CSA-13 exhibits antimicrobial activity against cariogenic and periodontopathic bacteria. Oral Microbiol. Immunol..

[145] Blasi F., Page C., Rossolini G.M., Pallecchi L., Matera M.G., Rogliani P., Cazzola M. (2016). The effect of N-acetylcysteine on biofilms: Implications for the treatment of respiratory tract infections. Respir. Med..

[146] Wang J., Ghali S., Xu C., Mussatto C.C., Ortiz C., Lee E.C., Tran D.H., Jacobs J.P., Lagishetty V., Faull K.F. (2018). Ceragenin CSA13 Reduces Clostridium difficile Infection in Mice by Modulating the Intestinal Microbiome and Metabolites. Gastroenterology.

[147] Aksit Bıcak D., Akyuz S., Kıratlı B., Usta M., Urganci N., Alev B., Yarat A., Sahin F. (2017). The investigation of Helicobacter pylori in the dental biofilm and saliva samples of children with dyspeptic complaints. BMC Oral Health.

[148] Abdul N.S., Khalid Alkhelaiwi A., Awadh Alenazi A., Fehaid Alrashidi R., Ghaleb Salma R. (2023). The Association of Helicobacter pylori in the Oral Cavity with Dental Caries in Patients with and Without Gastric Infection: A Systematic Review. Cureus.

[149] Leszczyńska K., Namiot A., Fein D.E., Wen Q., Namiot Z., Savage P.B., Diamond S., Janmey P.A., Bucki R. (2009). Bactericidal activities of the cationic steroid CSA-13 and the cathelicidin peptide LL-37 against Helicobacter pylori in simulated gastric juice. BMC Microbiol..

[150] Moscoso M., Esteban-Torres M., Menéndez M., García E. (2014). In vitro bactericidal and bacteriolytic activity of ceragenin CSA-13 against planktonic cultures and biofilms of Streptococcus pneumoniae and other pathogenic streptococci. PLoS ONE.

[151] Hodak C.R., Bescucci D.M., Shamash K., Kelly L.C., Montina T., Savage P.B., Inglis G.D. (2023). Antimicrobial Growth Promoters Altered the Function but Not the Structure of Enteric Bacterial Communities in Broiler Chicks ± Microbiota Transplantation. Animals.

[152] Vila T., Rizk A.M., Sultan A.S., Jabra-Rizk M.A. (2019). The power of saliva: Antimicrobial and beyond. PLoS Pathog..

[153] Do T., Devine D., Marsh P.D. (2013). Oral biofilms: Molecular analysis, challenges, and future prospects in dental diagnostics. Clin. Cosmet. Investig. Dent..

[154] Bucki R., Namiot D.B., Namiot Z., Savage P.B., Janmey P.A. (2008). Salivary mucins inhibit antibacterial activity of the cathelicidin-derived LL-37 peptide but not the cationic steroid CSA-13. J. Antimicrob. Chemother..

[155] Abouassi T., Hannig C., Mahncke K., Karygianni L., Wolkewitz M., Hellwig E., Al-Ahmad A. (2014). Does human saliva decrease the antimicrobial activity of chlorhexidine against oral bacteria?. BMC Res. Notes.

[156] Spijkervet F.K., van Saene J.J., van Saene H.K., Panders A.K., Vermey A., Fidler V. (1990). Chlorhexidine inactivation by saliva. Oral Surg. Oral Med. Oral Pathol..

[157] Leszczyńska K., Namiot A., Cruz K., Byfield F.J., Won E., Mendez G., Sokołowski W., Savage P.B., Bucki R., Janmey P.A. (2011). Potential of ceragenin CSA-13 and its mixture with pluronic F-127 as treatment of topical bacterial infections. J. Appl. Microbiol..

[158] Tokajuk J., Deptuła P., Chmielewska S.J., Skłodowski K., Mierzejewska Ż., Grądzka-Dahlke M., Tołstoj A., Daniluk T., Paprocka P., Savage P.B. (2022). Ceragenin CSA-44 as a Means to Control the Formation of the Biofilm on the Surface of Tooth and Composite Fillings. Pathogens.

[159] Li X., Kolltveit K.M., Tronstad L., Olsen I. (2000). Systemic diseases caused by oral infection. Clin. Microbiol. Rev..

[160] Symonds N.E., Meng E.X.M., Boyd J.G., Boyd T., Day A., Hobbs H., Maslove D.M., Norman P.A., Semrau J.S., Sibley S. (2024). Ceragenin-coated endotracheal tubes for the reduction of ventilator-associated pneumonia: A prospective, longitudinal, cross-over, interrupted time, implementation study protocol (CEASE VAP study). BMJ Open.

[161] Bucki R., Niemirowicz K., Wnorowska U., Byfield F.J., Piktel E., Wątek M., Janmey P.A., Savage P.B. (2015). Bactericidal activity of ceragenin CSA-13 in cell culture and an animal model of peritoneal infection. Antimicrob. Agents Chemother..

[162] Suphasiriroj W., Mikami M., Shimomura H., Sato S. (2013). Specificity of antimicrobial peptide LL-37 to neutralize periodontopathogenic lipopolysaccharide activity in human oral fibroblasts. J. Periodontol..

[163] McCrudden M.T.C., O’Donnell K., Irwin C.R., Lundy F.T. (2018). Effects of LL-37 on Gingival Fibroblasts: A Role in Periodontal Tissue Remodeling?. Vaccines.

[164] Liu T., Chen Y.C., Jeng S.L., Chang J.J., Wang J.Y., Lin C.H., Tsai P.F., Ko N.Y., Ko W.C., Wang J.L. (2023). Short-term effects of Chlorhexidine mouthwash and Listerine on oral microbiome in hospitalized patients. Front. Cell. Infect. Microbiol..

[165] Starr C.G., Wimley W.C. (2017). Antimicrobial peptides are degraded by the cytosolic proteases of human erythrocytes. Biochim. Biophys. Acta Biomembr..

[166] Chen C.H., Lu T.K. (2020). Development and Challenges of Antimicrobial Peptides for Therapeutic Applications. Antibiotics.

[167] Greco I., Molchanova N., Holmedal E., Jenssen H., Hummel B.D., Watts J.L., Håkansson J., Hansen P.R., Svenson J. (2020). Correlation between hemolytic activity, cytotoxicity and systemic in vivo toxicity of synthetic antimicrobial peptides. Sci. Rep..

[168] Blomstrand E., Posch E., Stepulane A., Rajasekharan A.K., Andersson M. (2024). Antibacterial and Hemolytic Activity of Antimicrobial Hydrogels Utilizing Immobilized Antimicrobial Peptides. Int. J. Mol. Sci..

[169] Wnorowska U., Fiedoruk K., Piktel E., Prasad S.V., Sulik M., Janion M., Daniluk T., Savage P.B., Bucki R. (2020). Nanoantibiotics containing membrane-active human cathelicidin LL-37 or synthetic ceragenins attached to the surface of magnetic nanoparticles as novel and innovative therapeutic tools: Current status and potential future applications. J. Nanobiotechnol..

[170] Niemirowicz K., Surel U., Wilczewska Z.A., Mystkowska J., Piktel E., Gu X., Namiot Z., Kułakowska A., Savage P.B., Bucki R. (2015). Bactericidal activity and biocompatibility of ceragenin-coated magnetic nanoparticles. J. Nanobiotechnol..

[171] Paprocka P., Durnaś B., Mańkowska A., Skłodowski K., Król G., Zakrzewska M., Czarnowski M., Kot P., Fortunka K., Góźdź S. (2021). New β-Lactam Antibiotics and Ceragenins—A Study to Assess Their Potential in Treatment of Infections Caused by Multidrug-Resistant Strains of. Infect. Drug Resist..

[172] Casciaro B., Dutta D., Loffredo M.R., Marcheggiani S., McDermott A.M., Willcox M.D., Mangoni M.L. (2017). Esculentin-1a derived peptides kill Pseudomonas aeruginosa biofilm on soft contact lenses and retain antibacterial activity upon immobilization to the lens surface. Biopolymers.

[173] Hashemi M.M., Holden B.S., Taylor M.F., Wilson J., Coburn J., Hilton B., Nance T., Gubler S., Genberg C., Deng S. (2018). Antibacterial and Antifungal Activities of Poloxamer Micelles Containing Ceragenin CSA-131 on Ciliated Tissues. Molecules.

[174] Skłodowski K., Chmielewska S.J., Depciuch J., Deptuła P., Piktel E., Daniluk T., Zakrzewska M., Czarnowski M., Cieśluk M., Durnaś B. (2021). Ceragenin-Coated Non-Spherical Gold Nanoparticles as Novel Candidacidal Agents. Pharmaceutics.

[175] Spałek J., Daniluk T., Godlewski A., Deptuła P., Wnorowska U., Ziembicka D., Cieśluk M., Fiedoruk K., Ciborowski M., Krętowski A. (2021). Assessment of Ceragenins in Prevention of Damage to Voice Prostheses Caused by. Pathogens.

